# Chronic silencing of subsets of cortical layer 5 pyramidal neurons has a long‐term influence on the laminar distribution of parvalbumin interneurons and the perineuronal nets

**DOI:** 10.1111/joa.14181

**Published:** 2024-12-03

**Authors:** Florina P. Szabó, Veronika Sigutova, Anna M. Schneider, Anna Hoerder‐Suabedissen, Zoltán Molnár

**Affiliations:** ^1^ Department of Physiology, Anatomy and Genetics, Sherrington Building University of Oxford Oxford UK; ^2^ Department of Stem Cell Biology Universitätsklinikum Erlangen/Friedrich‐Alexander‐Universität Erlangen‐Nürnberg Erlangen Germany; ^3^ Department of Neurology University Hospital Zürich and University of Zürich Zürich Switzerland

**Keywords:** cerebral cortex, GABAergic interneurons, layer 5, parvalbumin, perineuronal nets, Rbp4‐Cre, Snap25, synaptic transmission

## Abstract

Neural networks are established throughout cortical development, which require the right ratios of glutamatergic and GABAergic neurons. Developmental disturbances in pyramidal neuron number and function can impede the development of GABAergic neurons, which can have long‐lasting consequences on inhibitory networks. However, the role of deep‐layer pyramidal neurons in instructing the development and distribution of GABAergic neurons is still unclear. To unravel the role of deep‐layer pyramidal neuron activity in orchestrating the spatial and laminar distribution of parvalbumin neurons, we selectively manipulated subsets of layer 5 projection neurons. By deleting *Snap25* selectively from Rbp4‐Cre + pyramidal neurons, we abolished regulated vesicle release from subsets of cortical layer 5 projection neurons. Our findings revealed that chronically silencing subsets of layer 5 projection neurons did not change the number and distribution of parvalbumin neurons in the developing brain. However, it did have a long‐term impact on the laminar distribution of parvalbumin neurons and their perineuronal nets in the adult primary motor and somatosensory cortices. The laminar positioning of parvalbumin neurons was altered in layer 4 of the primary somatosensory cortex in the adult *Snap25* cKO mice. We discovered that the absence of layer 5 activity only had a transient effect on the soma morphology of striatal PV neurons during the third week of postnatal development. We also showed that the relationship between parvalbumin neurons and the perineuronal nets varied across different cortical layers and regions; therefore, their relationship is far more complex than previously described. The current study will help us better understand the bidirectional interaction between deep‐layer pyramidal cells and GABAergic neurons, as well as the long‐term impact of interrupting pyramidal neuron activity on inhibitory network formation.

## Graphical abstract

Chronic silencing of subsets of layer 5 projection neurons has distinct effects on the laminar distribution of PV interneurons and the perineuronal nets in the adult primary motor and somatosensory cortex.

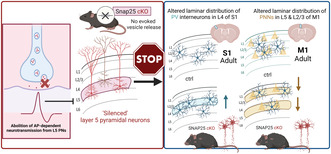

## 1 INTRODUCTION

The perplexing complexity of cortical circuits arises from the morphologically and functionally distinct cell types, which are represented across the six layers of the neocortex and proven to have unique input and output connections. These intricately designed networks in the cerebral cortex rely on the precise ratio of excitatory pyramidal neurons and inhibitory GABAergic neurons that provide domain‐specific innervation to distinct subclasses of pyramidal cells defined by their areal, laminar and long‐range projection targets (Kawaguchi, 1997; Kepecs & Fishell, 2014; Markram et al., 2004; Somogyi et al., 1998; Tremblay et al., 2016). Molecularly distinct subtypes of inhibitory interneurons play a pivotal role in shaping circuit functions, gating information flow to the cerebral cortex and synchronising the activity of neural assemblies in oscillatory neuronal networks (Gupta, 2000; McBain & Fisahn, 2001; Somogyi & Klausberger, 2004). In rodents, the GABAergic and glutamatergic neurons are generated in different sectors of the neuroepithelium under different combinatorial transcription codes (Butt et al., 2008; Marín & Rubenstein, 2001; Price et al., 1992; Sultan et al., 2013, Lodato & Arlotta, 2015). Disrupting the transcriptional identity of intratelencephalic (IT) projection neurons by knocking out the transcription factor *Satb2* from pyramidal neurons not only converted the IT‐type excitatory neurons to pyramidal‐tract (PT) type neurons but also caused aberrations in the cortical lamination of caudal ganglionic eminence (CGE)‐derived interneurons (Wester et al., 2019). Similarly, loss of the transcription factor *Fezf2* defining the fate of subcerebral projection neurons led to an aberrant laminar distribution of parvalbumin (PV) and somatostatin (SST) interneurons along with severe defects in GABAergic inhibition (Lodato et al., 2011). These studies suggest that both the transcriptional and the laminar identity of pyramidal neurons form an essential part of the cortical cytoarchitecture and may instruct the integration of GABAergic neurons into cortical circuits.

Although several subtypes of GABAergic neurons have long been studied for their ability to regulate pyramidal cell activity, less attention has been paid to the role that pyramidal neurons play in the establishment of inhibitory circuits. However, studies have emerged where perturbations in pyramidal cell function or local ablation of pyramidal cells have directly affected the number, survival and synaptic connectivity of GABAergic interneurons (Duan et al., 2020; Lodato et al., 2011; Sreenivasan et al., 2022; Wong et al., 2018, 2022). Interneuron survival in the nascent cerebral cortex is dependent on the neuronal activity of pyramidal cells that rescue them from undergoing apoptosis by strengthening their connections to GABAergic cells before the programmed cell death (Denaxa et al., 2018; Priya et al., 2018; Sreenivasan et al., 2022; Wong et al., 2018, 2022). The interdependency of neuronal signalling in glutamatergic pyramidal neurons and the survival of GABAergic neurons points to the role of pyramidal neuron activity in the assembly of inhibitory cortical circuits. The intrinsically programmed mechanism of interneuron apoptosis itself can be modulated by chemogenetically activating pyramidal neurons and/or inhibiting PTEN signalling in pyramidal cells during the critical window of interneuron cell death in mice (Southwell et al., 2012; Wong et al., 2018). When pyramidal neurons are temporarily activated chemogenetically, parvalbumin and somatostatin interneuron density rises; conversely, when pyramidal neuron activity is manipulated during crucial stages of postnatal development, it decreases with transient inhibition. (Wong et al., 2018). While these studies have primarily focused on the regulatory role of pyramidal neurons on the survival of cortical GABAergic interneurons, new evidence suggests that PV interneuron survival in subcortical projection regions of cortical pyramidal neurons depends on the presence of glutamatergic inputs. When excitatory inputs from corticostriatal layer 5 (L5) projection neurons were absent, the number of striatal interneurons declined (Sreenivasan et al., 2022). This further supports the role of glutamatergic transmission and cortical control over the cortical and subcortical subtypes of PV interneurons. Ablation of the vesicular glutamate transporters of Vglut1 and Vglut2 to eliminate glutamate release from pyramidal neurons has been shown to have a differential impact on the number of GABAergic interneurons. While the abolition of excitatory transmission from pyramidal neurons has caused a marked decrease in the density of neurogliaform and basket cells, it has not altered the density of bipolar neurons (Wong et al., 2022). These findings indicate that glutamatergic transmission and excitatory pyramidal neuron inputs govern the survival of inhibitory cells in the nascent brain. When migrating GABAergic neurons are devoid of glutamatergic inputs due to the pharmacological blockade of NMDA receptors, inhibition of excitatory neurotransmission has a deleterious influence on immature interneurons and promotes apoptotic death (Roux et al., 2015). Similarly, tetanus toxin injections to ablate excitatory inputs in the somatosensory cortex at postnatal day 0 (P0) caused a notable reduction in the number of somatostatin interneurons in the infragranular cortical layers at P9 (Duan et al., 2020). Intriguingly, the lack of elimination of the excess number of GABAergic neurons during development results in permanent disruptions that only surface in adulthood (Magno et al., 2021). Given the causal observation that the removal of excitatory synaptic inputs during neonatal development at P0 results in interneuron cell death, pyramidal cell activity may act as a key regulator in the emergence of GABAergic circuits.

Studies investigating the role of individual fusion proteins of the SNARE (soluble N‐ethylmaleimide fusion protein attachment protein receptor) machinery have confirmed that synaptosomal associated protein 25 kDa (SNAP25)‐executed membrane fusion and the regulation of voltage‐gated calcium channels are central to synaptic homeostasis and optimal brain functioning. We have previously shown that ablating *Snap25* from L5 and L6 projection neurons impacts myelination by reducing the length of the node of Ranvier and the g‐ratio in the dorsal column of the spinal cord (Korrell et al., 2019). We have also discovered that the presence of regulated vesicle release is fundamental to the maturation of the specialised synapses of L5 corticothalamic projection neurons in the posterior thalamic nucleus (Hayashi et al., 2021) and L5 plays an important part in the regulation of sleep–wake cycles (Krone et al., 2021). It remains to be elucidated; however, how perturbations in pyramidal cell activity impact GABAergic neurons both short‐term and long‐term. Specifically, how the main output projection neurons of the cortex in layer 5 regulate the distribution of GABAergic neurons in the nascent and the adult brain via activity‐dependent mechanisms.

To unveil the role of deep‐layer pyramidal neuron activity in orchestrating the spatial and laminar distribution of inhibitory neurons, we selectively manipulated infragranular projection neurons in layer 5 of the cortex. By ablating *Snap25* from selected subsets of glutamatergic L5 projection neurons across the cortical mantle, we abolished Ca^2+^−dependent vesicle release from Rbp4‐Cre neurons and chronically ‘silenced’ L5 projection neurons. The *Rbp4‐Cre*;*Ai14*;*Snap25* ^*fl/fl*^ conditional knockout mice (cKO) allowed us to selectively disrupt Ca^2+^‐dependent neurotransmission in infragranular pyramidal neurons while leaving spontaneous and constitutive vesicle release intact (Washbourne et al., 2002). We have previously shown that 15% of NeuN+ cells in L5 were labelled in the primary somatosensory cortex of the *Rbp4‐Cre*;*Ai14* mice (Hoerder‐Suabedissen et al., 2019). The long‐term manipulation of this subpopulation of layer 5 neurons produced changes in the maintenance of myelinated fibres and the establishment of specialised synapses (Hayashi et al., 2021; Korrell et al., 2019), as well as a noticeable behavioural phenotype (Krone et al., 2021). Moreover, this genetic manipulation has proven to be sufficient to have a drastic impact on the projections and survival of Rbp4‐Cre + neurons in adulthood (Hoerder‐Suabedissen et al., 2019).

We also documented that the presynaptically silenced deep‐layer projection neurons follow the same developmental dynamics as those of the control brains until P21 (Hoerder‐Suabedissen et al., 2019). Since no developmental abnormalities have been noticed, the cKO mice thus allow for the genuine investigation of activity‐dependent mechanisms on developing GABAergic circuits in a layer‐specific fashion, without the confounder of altered pyramidal cell number or genetic identity. After the first 3 weeks of development; however, *Snap25* cKO mice exhibit signs of degeneration including impaired axonal integrity and altered synapses, and there is further evidence for altered ultrastructure of neurites, progressive axonal degeneration and accumulation of inflammation markers in the *Snap25*‐ablated adult brains (Hoerder‐Suabedissen et al., 2019). The neurodegenerative processes are only apparent in adult L5‐silenced mice; therefore, we could examine how the synaptic activity of L5 impacted the spatial and laminar arrangement of GABAergic neurons *temporarily* (postnatal periods when L5 neurons are intact) and *permanently* (adult stages when L5 neurons start to degenerate). We also distinguished between the *local* (location of the cell bodies) and *global* (subcortical projection sites) effects of chronically silencing cortical L5 neurons on parvalbumin interneurons.

We discovered that the chronic abolition of evoked vesicle release from L5 projection neurons from the time shortly before birth reorganised the laminar distribution of cortical PV neurons in layer 4 of S1 in the adult brain. Interestingly, this effect was exclusive to the adult cortex suggesting a long‐term impact of silencing L5 on the laminar profile of PV cells. Abolishing regulated vesicle release from L5 revealed additional alterations in the laminar positioning of the different subpopulations of PV neurons in L5 (those with and without PNNs), which affected the density of perineuronal nets in the adult motor cortex. Only at P21 did the lack of L5 activity affect the soma features of striatal PV neurons temporarily, as the effect was rescued by adulthood. We found that the correlation between PV and *Vicia villosa* agglutinin‐labelled (VVA) neurons is influenced by cortical areas and layers, and the interaction between PV interneurons and perineuronal nets is far more complex than previously thought. Our findings demonstrate that Ca^2+^‐dependent synaptic neurotransmission from L5 projection neurons does not impede PV neuron development, but it has a persistent impact on the laminar arrangement of PV neurons, which only manifests in the adult cortex.

## 2 METHODS

### 2.1 Breeding and maintenance of transgenic mice

All experimental procedures were conducted in compliance with the project and personal licenses, as per the rules and regulations of the Animals (Scientific Procedures) Act 1986. The animal work was performed at the Biomedical Services (BMS) of the University of Oxford while adhering to the local regulations related to animal care and welfare. Animals were kept on a 12‐h light/12‐h dark cycle and food and water were available ad libitum. Transgenic mice with a genetic ablation in the synaptosomal associated protein 25 kDa (SNAP25) gene (*B6*‐*Snap25tm3mcw* (*Snap25*‐flox)) were used to engineer a cell‐type specific conditional knockout strain enabling the selective abolition of Ca^2+^‐dependent neurotransmitter release from subsets of glutamatergic projection neurons. The generation and characterisation of the *Rbp4*‐*Cre+*;*Ai14*;*Snap25* ^*fl/fl*^ mice and the validation of the absence of regulated vesicle release have previously been described (Gustus et al., 2018; Hayashi et al., 2021; Hoerder‐Suabedissen et al., 2019; Korrell et al., 2019; Krone et al., 2021; Marques‐Smith et al., 2016; Welch et al., 2000).

### 2.2 Cell‐type specific deletion of *Snap25* from subsets of glutamatergic cortical layer 5 projection neurons

To abolish regulated synaptic vesicle release from subsets of deep‐layer glutamatergic neurons, homozygous *B6‐Snap25tm3mcw* (*Snap25* ^*fl/fl*^) mice were first crossed to *B6*;*129S6*‐*Gt*(*ROSA*)*26Sortm14*(*CAG‐tdTomato*)*Hze/J* (*Ai14*) mice. The homozygous *Snap25* ^*fl/fl*^;*Ai14* mice were then crossed to a bacterial artificial chromosome (BAC) Cre‐recombinase driver line Tg(Rbp4‐cre)KL100Gsat/Mmucd (Rbp4‐Cre; Jackson Laboratories) suitable for the investigation of cortical pyramidal neurons in a layer‐specific manner. *Cre*/+;*Snap25* ^*fl/+*^;*Ai14* female mice were crossed to *Snap25* ^*fl/fl*^;*Ai14* males to generate conditional knockout (*Cre*+;*Ai14*;*Snap25* ^*fl/fl*^) and heterozygous knockout (*Cre+;Ai14;Snap25* ^*fl/+*^) mice. No heterozygous knockout mice were used in the experiments. *Cre*‐;*Ai14*;*Snap25* ^*fl/+*^ and *Cre*‐;*Ai14*;*Snap25* ^*fl/fl*^ were used as control animals. Note that the control mice lack tdTomato expression and therefore, no red fibres are visible in the control animals. We documented the fibre distribution in previous studies in *Rbp4‐Cre*;*Ai14* mice (Grant et al., 2016; Hoerder‐Suabedissen et al., 2019). Both male and female mice were used throughout this study. For the developmental studies on the effects of abolishing evoked vesicle release from L5 projection neurons, mice were perfused at postnatal day 14 (P14) and postnatal day 21 (P21). This captures the developmental stages when spontaneous neural activity shapes the emergence of inhibitory cortical circuits. For the adult studies, mice of both sexes were collected at 12 weeks of age where *Snap25* cKO mice already exhibited an accumulation of inflammation markers and signs of axonal degeneration including impaired axonal integrity and altered synapses (Hoerder‐Suabedissen et al., 2019; Vadisiute et al., 2024). For the number of animals used in the study, please see Table S1. For the selected cortical and subcortical regions of interest to distinguish between the local and global effects of L5 on PV neurons, please see Table S3.

### 2.3 Perfusion fixation and vibratome sectioning

Mice aged P14, P21 and 3 months of age were anaesthetised with an overdose of sodium pentobarbital (60 mg/kg, intraperitoneal injection (i.p.)) and were trans‐cardially perfused using ice‐cold saline (0.9% NaCl) solution followed by 4% paraformaldehyde (PFA, F8775; Sigma‐Aldrich) diluted in 0.1 M phosphate‐buffered saline (PBS, pH 7.4) (*n* = 3 brains per genotype for P14 experiments (3 control, 3 cKO), *n* = 4 brains per genotype for P21 experiments (4 control, 4 cKO) and *n* = 5 brains per genotype (5 control, 5 cKO) for the 12‐week‐old adult studies). Brains were dissected and postfixed in 4% PFA for 24 h at 4°C. Following post‐fixation, brains were transferred to 0.1 M PBS containing 0.05% sodium azide (PBSA) (Sodium azide ReagentPlus®, S2002‐5G, Sigma‐Aldrich, CAS number: 26628–22‐8) and kept at 4°C for long‐term storage. Coronal slices of 50 μm thickness were cut from 4.5% agarose‐embedded brains using a vibrating microtome (Leica VT1000S; Leica Microsystems, Wetzlar, Germany) and hemisections were collected in 0.1 M PBSA in 24‐well cell culture plates (CLS3526, Corning® Costar®).

### 2.4 Histology

#### 2.4.1 Labelling parvalbumin interneurons and the perineuronal nets

To study how the abolition of Ca^2+^‐dependent neurotransmission from selective subsets of L5 pyramidal neurons affects the density of cortical and subcortical GABAergic neurons, double histochemistry (IHC) was performed to label parvalbumin (PV) interneurons and the perineuronal nets (PNNs) using the *Vicia villosa* agglutinin (VVA) plant lectin.

For free‐floating PV & VVA double histochemistry, sections were washed three times in 0.1 M PBS for 10 min. Sections were then permeabilised with a blocking solution containing 2% donkey serum (D9663, Sigma‐Aldrich) and 0.2% Triton‐X100 (X‐100, CAS number: 9002‐93‐1, Sigma‐Aldrich) diluted in VVA buffer for 2 h at room temperature (RT) prior to overnight incubation with primary antibody at 4°C. For Vglut1 staining, 5% goat serum and 0.3% Triton‐X100 were used. VVA buffer consisted of 0.01 M PBS, 0.15 M NaCl and 0.1 mM CaCl_2_ to reach the minimum Ca^2+^ level required for optimal lectin binding. Rabbit anti‐PV (Cat# PV27, RRID:AB_2631173, Swant, Marly, Switzerland) was diluted at 1:5000 for postnatal and 1:500 for adult brains, and the biotinylated VVA lectin (B‐1235‐2, Vector Laboratories, Burlingame, US) was used at 2 μg/mL concentration and diluted in a blocking solution consisting of 2% donkey serum and 0.2% Triton‐X100 diluted in VVA buffer. To ascertain whether Rbp4/tdTomato‐expressing axonal processes and Vglut1‐positive synapses colocalise in the striatum, slices were incubated with guinea pig anti‐Vglut1 (1:500, EMD Millipore AB5905, lot: 3258729). After rinsing sections three times in 0.1 M PBS, slices were incubated with the following secondary antibodies: goat anti‐guinea pig Alexa Fluor™ 633 (Invitrogen, A‐21105), donkey anti‐rabbit Alexa Fluor™ 488 (Invitrogen, A‐21206, Thermo Fisher, Waltham, MA USA 02451) at 1:500 and Streptavidin‐conjugated Cy5 (Invitrogen, SA1011) at 1:200 for 2 h at RT. Sections were rinsed two times in 0.1 M PBS for 10 min and counterstained with 4,6‐diamidine‐2‐phenylindole dihydrochloride (DAPI) (1:1000 in 0.1 M PBS, overnight). Postnatal coronal sections were coverslipped with 0.1 M PBS, while adult sections were coverslipped using Prolong™ Gold antifade mountant (P36930, Molecular Probes, Eugene, OR USA 97402). To determine the specificity of the antibody used, control sections were processed following the same steps of histochemistry, but they were incubated only with secondary antibodies. Non‐specific staining was not observed in any samples. When performing single immunofluorescence staining for PV, no VVA buffer was used, and sections were permeabilised with a blocking solution containing 2% donkey serum and 0.2% Triton‐X100 diluted in 0.1 M PBS for 2 h.

#### 2.4.2 Image acquisition

Images containing the cortical and subcortical targets of Rbp4‐Cre + L5 projection neurons were acquired with an inverted laser‐scanning confocal microscope (Zeiss LSM 710, Germany) equipped with the following laser lines: Diode (405 nm), Argon laser (458, 488, 514 nm), Diode‐Pumped Solid‐State (DPSS, 561 nm), Helium‐Neon (633 nm). High‐resolution confocal images for PV and VVA quantification were acquired using an EC Epiplan‐Apochromat 20× (0.80 NA) dry objective at 1× optical zoom, while low‐power photomicrographs representing the location of high‐power confocal images were obtained with an upright widefield fluorescence microscope equipped with a colour camera for brightfield and fluorescence imaging. (Leica DMR, DFC 280, HC PL FLUOTAR 2.5 × /0.07). For the density and laminar distribution analyses of cortical PV+ neurons, 1 × 4 tile scans spanning the whole depth of M1 and S1 were acquired using sequential (best signal) mode scanning. For the density and laminar distribution analyses of cortical VVA+ cells, 1 × 4 tile scans combined with z‐stack imaging were acquired. For subcortical region analyses, 20× confocal image stacks were obtained for PV and VVA quantification and Z‐projection images using the maximum intensity function in Fiji (ImageJ) were produced for subsequent cell quantification. For each channel, the optimal laser power, digital gain, offset and pinhole settings were identified and kept constant throughout image acquisition.

### 2.5 Image analysis

#### 2.5.1 Cell quantification of PV+ neurons

Confocal image files of ‘.lsm’ extensions were processed in Fiji (ImageJ). For the quantification of cortical PV+ interneurons in M1 and S1, a semi‐automated custom‐designed macro written in Fiji (NIH) was used to assess the density and laminar distribution of PV cells both in the postnatal and adult brains. Due to the varying nature of PV morphology in subcortical brain regions, PV+ cells were quantified manually using the Cell Counter plugin of Fiji (ImageJ) in the globus pallidus, the higher‐order thalamic nuclei and the superior colliculus. The accuracy of the semi‐automated cell quantification method was confirmed by manual recounting of cells. The workflow of the custom‐designed pipeline is as follows. In brief, multichannel confocal images were first duplicated and split into individual channels using the Fiji *Color ‐ > Split* Channels function. Pre‐processing of images was then conducted using the rolling ball algorithm to correct uneven backgrounds and a linear Gaussian blur filter with a sigma value of 1 was applied to reduce noise. After filtering and noise correction, segmentation of foreground (objects of interest) from background objects was performed using the Otsu thresholding method. Following segmentation, a Watershed binary process was applied to separate touching or merged objects. Finally, the *Analyse particle > Set measurements* function was used to set the size and circularity of objects (size = 40‐Infinity, circularity = 0.10–1.00). Objects falling outside the preset range were excluded from the cell measurements. Double‐positive PV+ VVA+ cells were identified based on the colocalisation of PV with the VVA channel and were manually counted.

#### 2.5.2 Cell quantification of VVA+ cells

For the density and laminar distribution analysis of VVA+ cells in the adult brain, manual cell counting was selected over semi‐automated quantification as the segmentation of the lattice‐like morphology of PNNs proved to be unreliable. Manual quantification of VVA+ cells was performed using the Cell Counter plugin of Fiji. VVA+ cells that exhibited a typical web‐ or lattice‐like morphology and colocalised with the nuclear counterstain DAPI signal were counted as positives. Histochemical staining of PNNs in the developing brains yielded varied results and therefore, the VVA+ cells were only quantified in the adult brain at 3 months of age. In the postnatal brains, the visualisation of PNNs was either insufficient due to low staining intensity or the staining produced diffuse non‐specific labelling in subcortical structures. The typical web or lattice‐like structure of PNNs was not detectable in all postnatal brains. For the purpose of quantifying the density of VVA+ cells in nascent brains, P14 and P21 brains were deemed inappropriate. Considering the condensation of proteoglycans into PPNs is not yet complete at such early developmental ages, the detection of PNNs and the quantification of VVA+ cells might not be feasible with plant lectins during early brain development.

#### 2.5.3 Morphometric analysis of PV interneurons

Point‐scanning confocal z‐stack images of the caudoputamen were obtained using a 20x objective (Epiplan‐Apochromat 20x (0.80 NA) dry) and maximum intensity projected in Fiji to construct 2D images for morphometric analysis of PV neurons. The analysis pipeline was identical to that of the density and laminar distribution analyses, and the *Analyse > Set measurements* function was used to select the parameters to be investigated, that is *soma area, soma circularity, soma perimeter, soma roundness and soma solidity*. Following the automated quantification of PV cells, the result of density counts and morphometric measurements were stored in ‘.csv’ files and exported to Prism 10 (GraphPad) for plotting and statistical analysis. Scatter plots of morphometric analyses of PV+ cells represent individual cell measurements. For the number of cells measured for morphometric analyses and the number of cells examined at different time points, please see Table S2.

### 2.6 Statistics

Cell density and laminar distribution analyses of PV+ and VVA+ were performed on data obtained from *n* = 3 brains per genotype for P14 experiments (3 control, 3 cKO), *n* = 4 brains per genotype for P21 experiments (4 control, 4 cKO) and *n* = 5 brains per genotype (5 control, 5 cKO) for the 12‐week‐old adult studies. Box plot values are represented as mean ± SEM. When comparing two groups (*morphometric analysis of PV+*), a two‐tailed unpaired t‐test with Welch's correction was applied. When comparing more than two groups (density, distribution and dynamics of PV+ and VVA+ cells), a two‐way ANOVA with Šídák's multiple comparisons test was used. Scatter plots of morphometric analyses of PV+ cells represent individual cell measurements. Heatmaps present the Pearson correlation between PV and VVA in different cortical regions and different cortical layers (PV, *n* = 5 per genotype; VVA, *n* = 4 per genotype). Statistical tests were computed in Prism 10.2.1 (GraphPad Software, San Diego, CA, USA).

## 3 RESULTS

### 3.1 The *Rbp4‐Cre;Ai14;Snap25* ^*fl/fl*^ mice genetically label two types of cortical cells – Subsets of cortical layer 5 projection neurons and hippocampal granule cells

**FIGURE 1:**
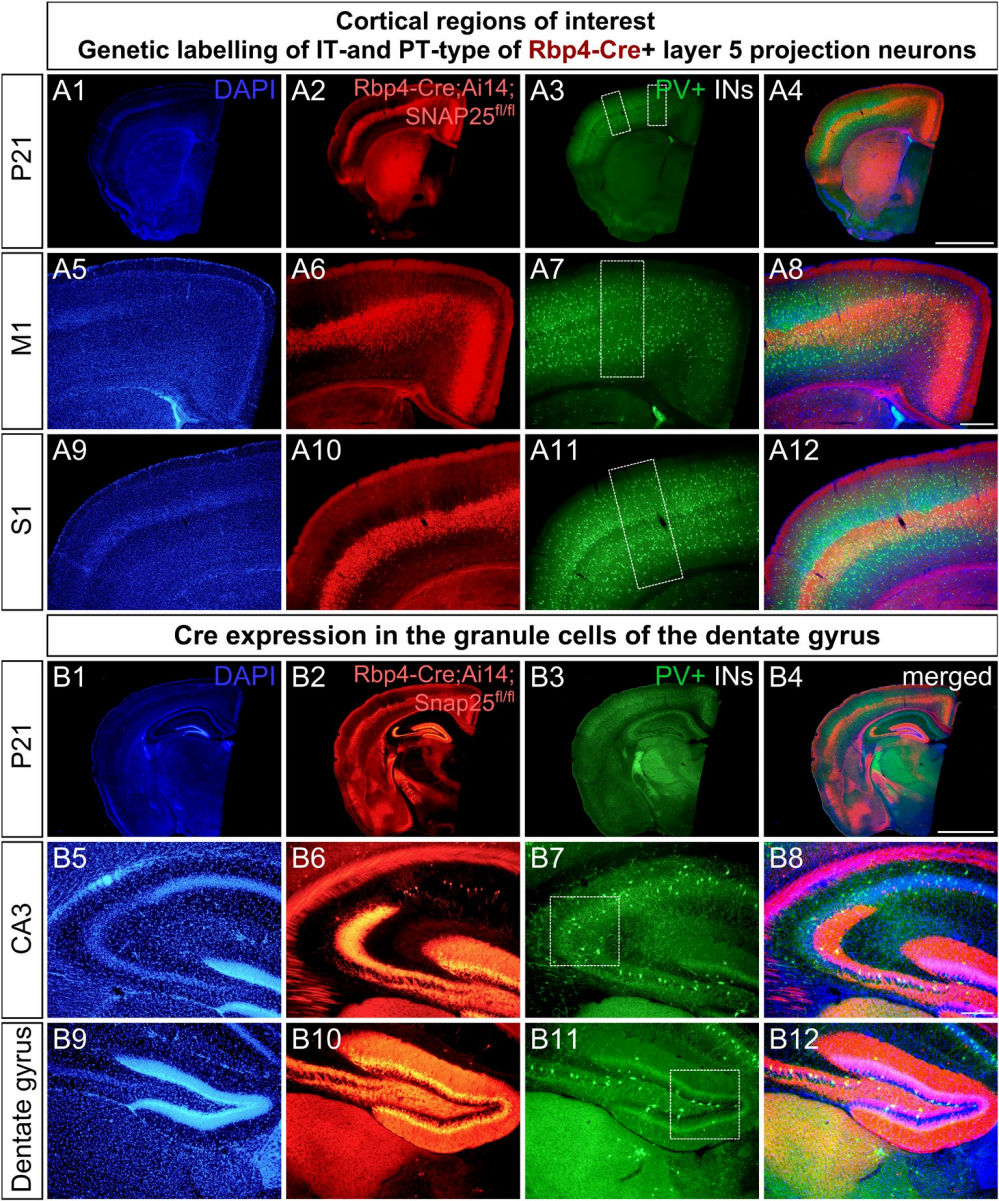
Distribution of parvalbumin interneurons and Rbp4‐Cre‐expressing neurons in layer 5 of the cortex and the dentate gyrus of the hippocampus in the *Snap25* cKO mice (*Rbp4‐Cre*;*Ai14*;*Snap25* ^*fl/fl*^). Low‐magnification epifluorescence photomicrographs of *Snap25* cKO brains at P21 (A1‐A4, B1‐B4) counterstained with DAPI (blue) and immunostained for parvalbumin neurons (green) (A3, A7, A11, B3, B7, B11). Epifluorescence images illustrating the distribution of silenced Rbp4‐Cre + projection neurons in the primary motor and somatosensory cortices (A2, A6, A10). Note that the Rbp4‐Cre driver line labels mixed subpopulations of intratelencephalic (IT) and extratelencephalic (ET) projection neurons in L5 of the cerebral cortex (A2, B2). Merging *Rbp4‐Cre*;*Ai14*; *Snap25* ^*fl/fl*^ and PV epifluorescence micrographs reveals the presence of PV‐immunoreactive neurons in the vicinity of the cell bodies of L5 projections neurons in the primary motor and somatosensory cortices (A4, A8, A12). Cortical regions of interest (ROIs) selected to study the local effects of abolishing evoked vesicle release from L5 pyramidal neurons on PV interneurons are marked by white boxes (A7, A11). Nuclear counterstains of cortical and hippocampal ROIs (A1, A5, A9, B1, B5, B9). Epifluorescence images depicting the distribution of Cre expression in the developing *Snap25* cKO brains in L5 across the cortical mantle and the hippocampus (B2, B4). Note the robust Cre expression in the granule cells of the dentate gyrus indicating the chronic silencing of the mossy fibre pathway (B6, B10). White boxes denote hippocampal ROIs, that is CA3 and dentate gyrus (B7, B11). Fluorescence micrographs of Cre‐expressing hippocampal granule cells superimposed on PV+ interneurons in the *Snap25* cKO mice (B4, B8, B12). Scale bars: 1000 μm (A4, B4), 200 μm (A8), 100 μm (B8).

To elucidate the role of subsets of glutamatergic layer 5 cortical projection neurons in the development and spatial organisation of GABAergic PV neurons, we conditionally deleted *Snap25* from Rbp4‐Cre + neurons in L5 of the cortex and selectively abolished calcium‐dependent vesicle release from specific subsets of L5 neurons. To distinguish between the *local* vs. *global* effects of L5 on the density and distribution of PV interneurons, we selected our regions of interest (ROI) based on the location of the cell bodies (*local effec*t) and the long‐range axonal projections of the silenced L5 *Snap25* cKO neurons (*global effect*). The primary motor (M1) and the primary somatosensory (S1) cortices are densely populated with Rbp4‐Cre + cortical projection neurons (Figure 1 A2, A6, A10) and PV interneurons (Figure 1 A3, A7, A11); therefore, we selected M1 and S1 as our cortical ROIs. In the *Rbp4*‐*Cre*+;*Ai14*;*Snap25* ^*fl/fl*^ mice, Cre expression is mostly detected in the pyramidal tract (PT) and intratelencephalic (IT) subgroups of L5 projection neurons throughout the neocortex. However, Cre‐expressing tdTomato+ neurons were also observed in the dentate gyrus (Figure 1 B2, B4, B6, B8, B10, B12) indicating that Ca^2+^‐dependent vesicle release was ablated in the granule cells in addition to the Rbp4‐Cre + L5 neurons. The *Snap25* cKO mice can thus be used not only for studying the effects of chronically silencing L5 projection neurons but also for the effects of chronically silencing the mossy fibre pathway on PV interneurons (Figure 1 B3, B7, B11). However, the hippocampal regions are not output regions of Rbp4‐Cre + L5 neurons and therefore, our analysis concentrated only on the long‐range projection sites of L5 neurons.

### 3.2 Chronically ‘silenced’ Rbp4‐Cre + glutamatergic projection neurons in layer 5 exhibit similar corticothalamic and corticofugal projections as Rbp4‐Cre + neurons

**FIGURE 2:**
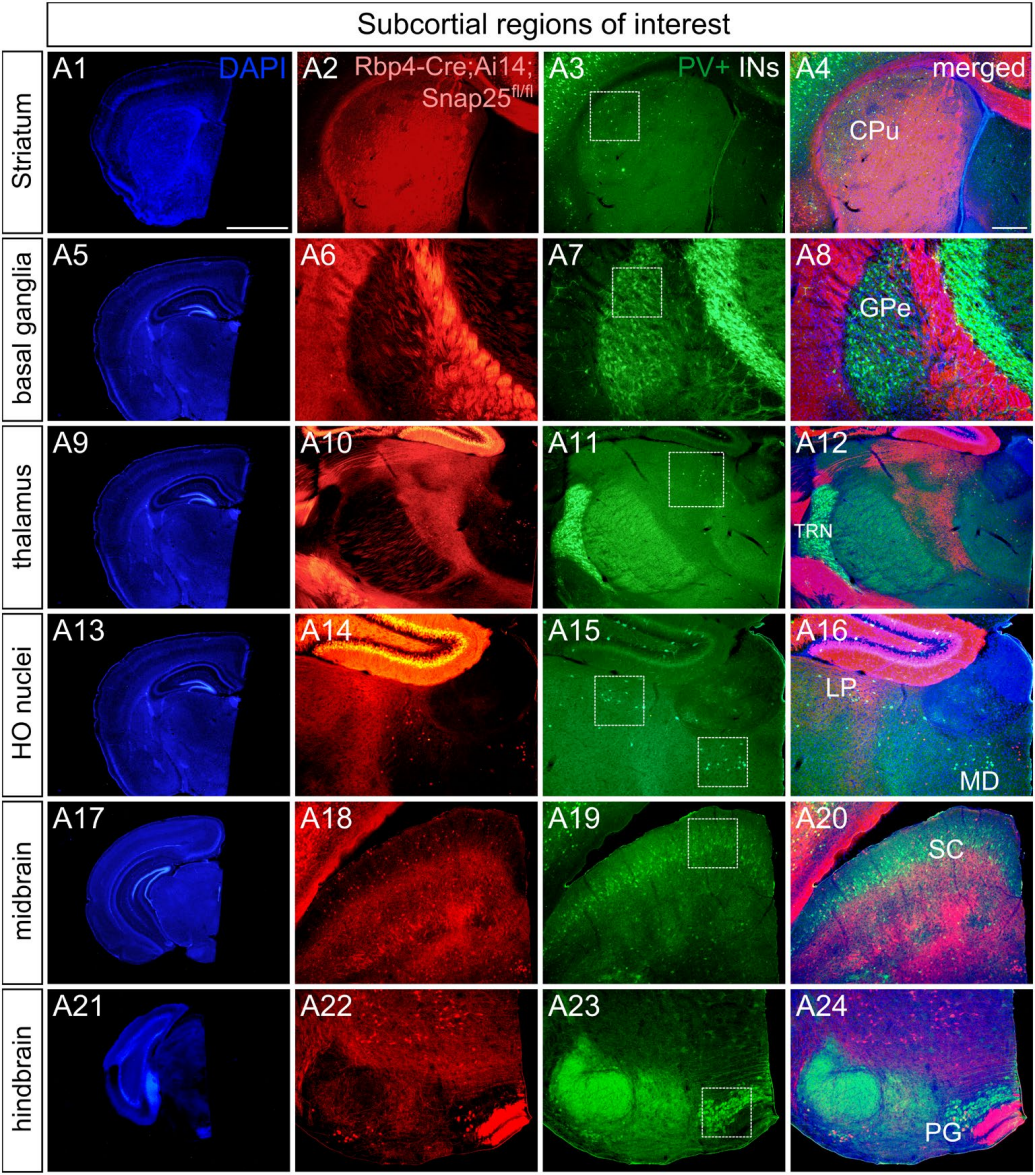
Long‐range axonal projections of the ‘silenced’ cortical layer 5 *Rbp4‐Cre*;*Ai14*;*Snap25* ^*fl/fl*^ neurons. Epifluorescence micrographs of coronal hemisections of P21 *Snap25* cKO mice obtained at different rostrocaudal levels and counterstained with DAPI (A1, A5, A9, A13, A17, A21). Long‐range corticothalamic and subcortical axonal projections of *Rbp4‐Cre*;*Ai14*; *Snap25* ^*fl/fl*^ neurons innervate the basal ganglia (A2, A6), the higher‐order thalamic nuclei (A10, A14) and the midbrain superior colliculus (A18). Collaterals of the descending corticofugal fibres of Rbp4‐Cre + L5 projection neurons target the brainstem motor regions including the pontine gray (A22) before reaching the spinal cord. White boxes define subcortical regions of interest (ROIs): The caudoputamen (A2‐A4), the external segment of the globus pallidus (A6‐A8), the lateral posterior nucleus of the thalamus (A10‐A12, A15‐16), the mediodorsal thalamic nucleus (A15–A16), the superior colliculus (A18‐A20) and the pontine gray (A22–A24). Specific subcortical projection sites of Rbp4‐Cre + L5 neurons were carefully selected to explore the global effects of abolishing Ca^2+^−dependent neurotransmission from L5 projection neurons. Note the distribution of subcortical PV neurons overlaps with the output regions of silenced L5 cortical projection neurons (A4, A8, A12, A16, A20, A24). Scale bars: 1000 μm (A1), 200 μm (A4). HO nuclei: Higher‐order nuclei.

To investigate if the chronic removal of Ca^2+^−dependent neurotransmission from L5 has a global effect on PV neurons, we selected those output regions of Rbp4‐Cre + tdTomato+ L5 neurons where PV‐immunoreactive cells were abundant (Figure 2 A3, A7, A11, A15, A19, A23). *Rbp4‐Cre;Snap25* ^*fl/fl*^ L5 neurons project to the contralateral caudoputamen (CPu) (Figure 2 A2, A4), the globus pallidus external segment (GPe) (Figure 2 A6, A8), the higher‐order thalamic nuclei including the lateral posterior nucleus (LP) (Figure 2 A10, A12) and the mediodorsal nucleus of the thalamus (MD) (Figure 2 A14, A16). Collaterals of the silenced Rbp4‐Cre L5 axons also target midbrain areas such as the pontine nuclei (Figure 2 A22, A24) and the superior colliculus (SC) (Figure 2 A18, A20), and they extend to the spinal cord through the pyramidal tract. Of the output regions of L5 *Rbp4*‐*Cre*;*Snap25* ^*fl/fl*^ neurons, the following innervation sites were selected as our subcortical ROIs: CPu, LP, MD, GPi and SC (Table S3). These subcortical regions are of great importance as the silenced subsets of pyramidal cells of layer 5 may exert a considerable effect on the number and distribution of PV+ cells through their long‐range corticofugal projections.

### 3.3 Chronic abolition of evoked vesicle release from subsets of layer 5 projection neurons does not affect the development of PV neurons in the second and third weeks of postnatal development

**FIGURE 3:**
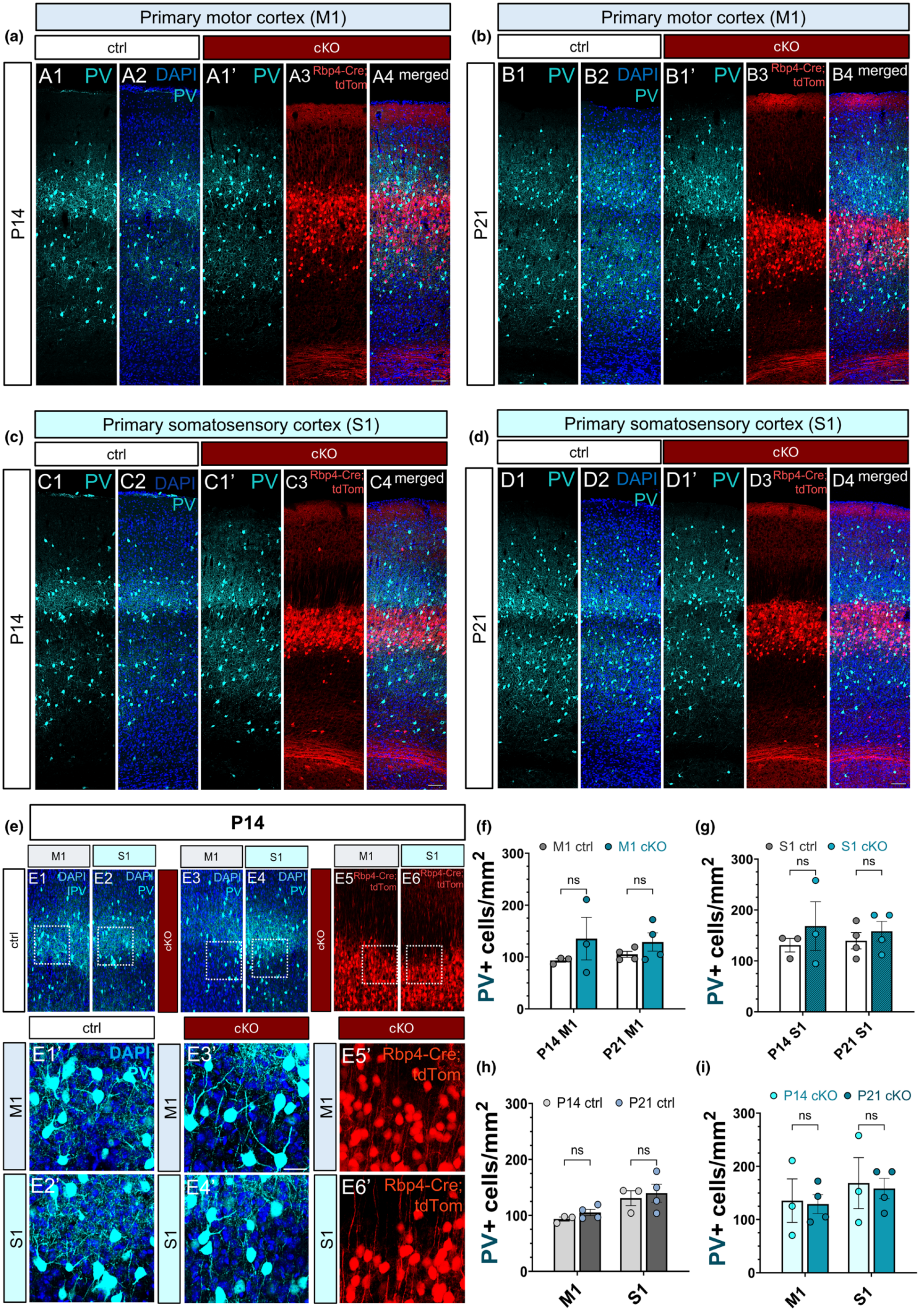
Perturbing the activity of Rbp4‐Cre + projection neurons in cortical layer 5 has no local effect on the density of cortical PV interneurons. 2D tile scans of point‐scanning confocal microscope images of the primary motor cortex during the second and third postnatal weeks of development. PV‐immunoreactive neurons (A1, A2, A1′, B1, B2, B1′) and *Rbp4‐Cre*;*Ai14*;*Snap25* ^*fl/fl*^ projection neurons in the L5‐silenced *Snap25* cKO brains in M1 at P14 (A3–A4) and P21 (B3–B4). Laser‐scanning confocal tile‐scan images of the primary somatosensory cortex depicting PV‐positive neurons and *Rbp4‐Cre*;*Ai14*;*Snap25* ^*fl/fl*^ projection neurons (C3‐C4, D3‐D4) in S1 in the control and *Snap25* cKO brains at P14 (C1, C2, C1′) and P21 (D1, D2, D1′). High‐magnification confocal images of PV interneurons in M1 and S1 in the control (E1, E2, E1′, E2′) and L5‐silenced brains at P14 (E3, E4, E3′, E4′). *Snap25*‐deficient cortical projection neurons in layer 5 of M1 and S1 at P14 (E5, E6, E5′, E6′). The regions of high‐magnification images are delineated by dotted white boxes (E1, E2, E3, E4). Quantification of PV cells in M1 in the second and third postnatal weeks revealed no significant differences in the density of PV neurons between the control and the chronically silenced L5 brains (f). No changes were detected in the density of PV neurons in S1 at P14 and P21 either (g). The developmental dynamics of cortical PV interneurons in the control brains revealed no significant changes in their density between P14 and P21 in the two cortical regions investigated (h). Abolition of regulated vesicle release from L5 pyramidal neurons did not alter the developmental dynamics of PV neurons (i). Scale bars: 100 μm (all panels). Data are represented as mean ± SEM. M1: Primary motor cortex; S1: Primary somatosensory cortex. (f–i), two‐way ANOVA with Šídák's multiple comparisons test.

To ascertain whether regulated synaptic vesicle release from L5 is required for the development of PV interneurons, we assessed the local effects of silencing Rbp4‐Cre + neurons on the density of cortical PV neurons at postnatal day 14 (P14) and postnatal day 21 (P21). No changes were detected in the density of PV‐immunoreactive cells in M1 at P14 and P21 (Figure 3f A1‐A1′, B1‐B1′, E1, E1′, E3, E3′). At both P14 and P21, the density of cortical PV neurons in S1 remained unaffected indicating the abolition of evoked neurotransmitter release from L5 pyramidal neurons does not influence the density of cortical PV+ cells (Figure 3g C1‐C1′, D1‐D1′, E2, E2′, E4, E4′). We next investigated the developmental dynamics of cortical PV interneurons during P14 and P21. Neither M1 nor S1 showed a change in PV cell density between P14 and P21 in the control brains suggesting that the number of PV interneurons following developmentally programmed cell death does not change significantly (Figure 3h). The developmental dynamics of cortical PV+ interneurons in the mutant brains were identical to the dynamics observed in the control brains (Figure 3i). These results suggest that the development of cortical PV interneurons is not influenced by the chronic abolition of neurotransmitter release from L5 projection neurons at P14 and P21.

### 3.4 Chronic abolition of evoked vesicle release from subsets of layer 5 projection neurons leaves the number of subcortical PV interneurons unchanged during development

**FIGURE 4:**
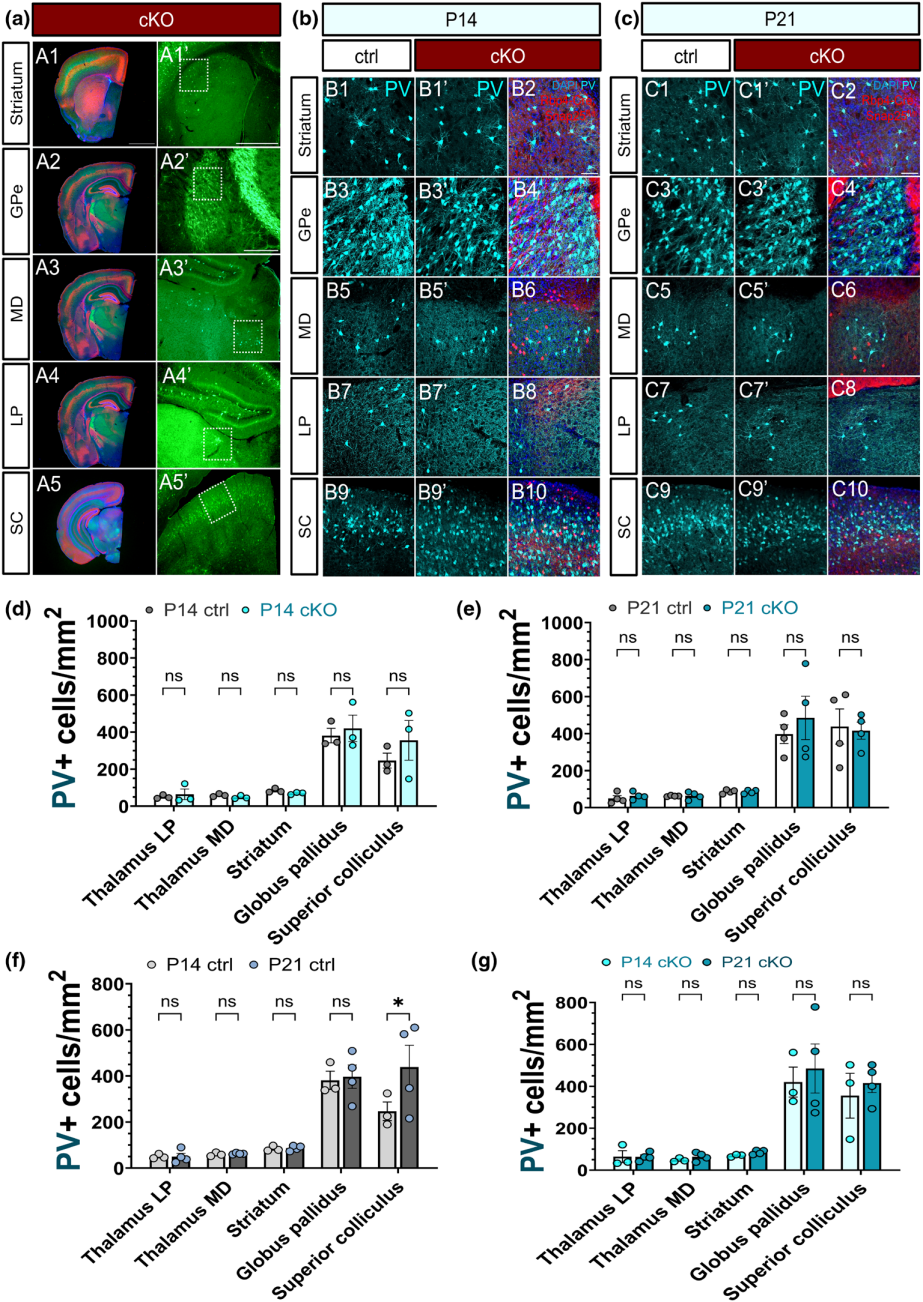
Perturbing the activity of Rbp4‐Cre + projection neurons in cortical layer 5 has no global impact on the density and dynamics of subcortical PV interneurons. Merged, low‐magnification epifluorescence images showing the level of sections selected for density analyses (A1, A2, A3, A4, A5). Epifluorescence micrographs illustrating subcortical regions of interest. The white dotted squares highlight the location of images presented in panels b and c (A1′, A2′, A3′, A4′, A5′). Maximum intensity projected z‐stack images representing the projection regions of cortical Rbp4‐Cre + L5 neurons at various rostrocaudal levels in the *Snap25* control and cKO brains in the second (B2, B4, B6, B8, B10) and third postnatal week (C2, C4, C6, C8, C10). The red signal indicates the axonal fibres of the ‘silenced’ layer 5 projection neurons. Parvalbumin‐immunoreactive neurons (cyan) shown in the caudoputamen (B1, B1′, C1, C1′) and the external segment of the globus pallidus (B3, B3′, C3, C3′) in the control and cKO brains at P14 (B1–B4) and P21 (C1–C4). PV+ neurons in the higher‐order lateral posterior and mediodorsal nuclei of the thalamus at P14 (B5‐B8) and P21 (C5–C8) which are selectively innervated by cortical L5 and L6b glutamatergic projection neurons. Maximum intensity projected z‐stack image showing PV‐immunoreactive neurons in the superior colliculus at P14 (B9–B10) and P21 (C9‐C10). Quantification of PV+ cell density in the subcortical projection regions of *Rbp4‐Cre*;*Ai14*;*Snap25* ^*fl/fl*^ cortical neurons at P14 (d) and P21 (e) revealed no significant differences in the density of PV+ neurons between the *Snap25* control and cKO brains. The developmental dynamics of subcortical PV neurons in the output regions of L5 neurons assessed between P14 and P21 in the control (f) and cKO (g) brains. The control brains showed a significant increase in the density of PV+ neurons in the superior colliculus (f) between P14 and P21, whereas no such change was observed in the L5‐silenced mice (g). Scale bars: 100 μm (A1), 500 μm (A1′), 200 μm (A2′). All data is represented as mean ± SEM values. *p < 0.05, SC, P14‐P21, ctrl, *p* = 0.0217. 2‐way ANOVA with Šídák's multiple comparisons test.

Next, we assessed the global effect of the chronic abolition of regulated vesicle release from L5 on subcortical PV neurons. At P14, none of the subcortical regions displayed a significant difference in the density of PV‐immunoreactive cells between the control and the *Snap25* cKO brains (Figure 4a,c A1‐10). Similarly, no alterations were found in the density of subcortical PV interneurons at P21 implicating the chronic cessation of layer 5 activity does not have a global influence on the development of PV neurons (Figure 4b,d B1–B10). Considering that the maturation of subcortical PV+ neurons may follow a different dynamic than that of the cortical PV+ cells, we next established the developmental profile of subcortical PV neurons. Of the output regions of L5 projection neurons, there was a significant increase in the density of PV+ cells in the superior colliculus between P14 and P21 in the control brains (Figure 4e). No changes were observed in the higher‐order thalamic nuclei and the basal ganglia (Figure 4e). As opposed to the significant rise noted in the control brains, PV cell density did not follow the same trend in the *Snap25* cKO mice between P14 and P21 (Figure 4f) indicating different developmental dynamics of PV neurons in the superior colliculus after L5 was silenced.

### 3.5 The chronic absence of synaptic vesicle release from Rbp4‐Cre + layer 5 pyramidal neurons alters the soma morphology of striatal PV+ neurons at P21

**FIGURE 5:**
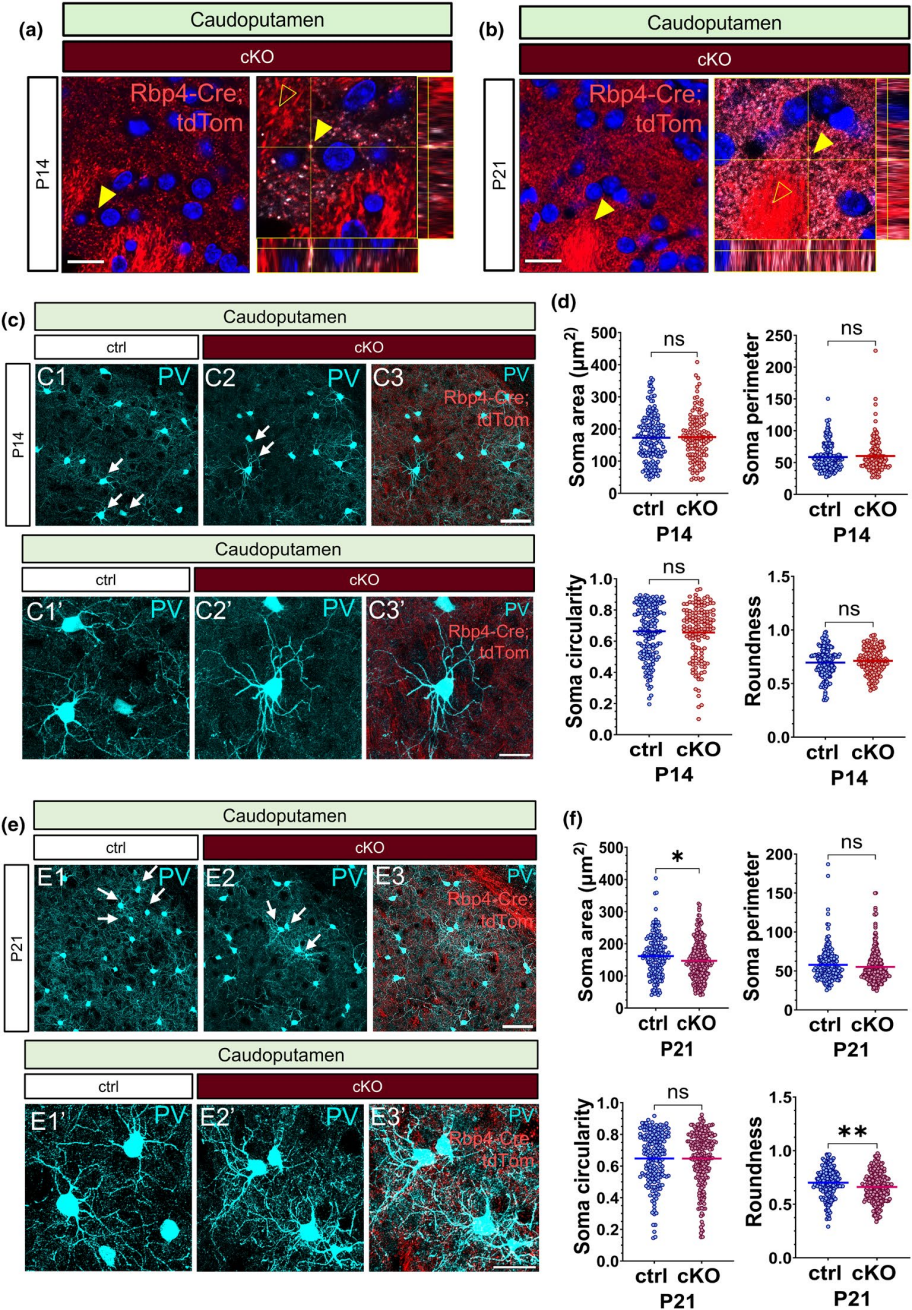
Abolition of Ca^2+^−dependent neurotransmission from layer 5 pyramidal neurons has a short‐term impact on the soma morphology of striatal PV interneurons at P21. Glutamatergic synapses are formed in the dorsal striatum by Rbp4‐tdTomato+ neurons (a, b). Confocal point‐scanning images of P14 and P21 *Rbp4‐Cre*;*Ai14;Snap25* ^*fl/fl*^ brains displaying puncta of VGluT1+ and tdTomato+ in the dorsal striatum in addition to orthogonal views through image stacks. Fluorescence colocalisation is evident at both ages (yellow arrowheads), with more punctae noted at P21 (white arrowheads). The tdTom+ fibre bundles (empty yellow arrowheads) are negative for VGluT1 staining (a, b). Maximum intensity projections of confocal z‐stack images of PV‐immunoreactive neurons in the caudoputamen at P14 and P21 in the control (C1, C1′, E1, E1′) and the L5‐silenced brains (C2, C2′, E2, E2′). Merged maximum intensity projected confocal images demonstrating the presence of tdTomato+ fibres in the caudoputamen in the vicinity of PV+ neurons (C3, C3′, E3, E3′). There were no significant alterations in the assessed soma features of PV+ neurons at P14 (d). Significant reductions in the soma area and the soma roundness of striatal PV+ neurons were detected at P21 in the *Snap25* cKO mice (f) (soma area, *p* = 0.0192; roundness, *p* = 0.0035, control, *n* = 180; cKO, *n* = 236 cells). Unpaired *t*‐test with Welch's correction. *p < 0.05, **p < 0.01. Scale bars: 50 μm (C3, E3, E3′), 20 μm (a, b, C3′).

After establishing that the chronic cessation of Ca^2+^‐dependent neurotransmission from Rbp4‐Cre + L5 projections neurons does not have a global influence on the density of subcortical PV+ neurons in its projection regions, we next investigated whether the disruption of L5 affects the morphology of PV cells in the striatum. This output region of L5 is not only innervated by the axonal projections of Rbp4‐Cre + neurons (Figure 5c C3, C3′, 9e E3, E3′) but also receives dense synaptic innervation as shown by the presence of Vglut1+ tdTomato+ punctae in the dorsal striatum (Figure 5a,b). No differences were detected between the control and the cKO brains at P14 in any of the measured soma parameters of striatal PV+ neurons (Figure 5c,d). However, at P21, we noticed a significant decrease in the soma area and roundness of striatal PV+ neurons in the L5‐silenced brains (Figure 5e,f). Although the chronic abolition of vesicle release from L5 did not alter the density and the developmental dynamics of striatal PV+ neurons at P21, it did modify their morphology. Striatal PV+ interneurons appeared to be more elongated, with less rounded and smaller somata in the layer 5‐silenced mice.

### 3.6 Striatal PV interneurons in the layer 5‐silenced brains display altered developmental dynamics of soma features between postnatal stages of development

**FIGURE 6:**
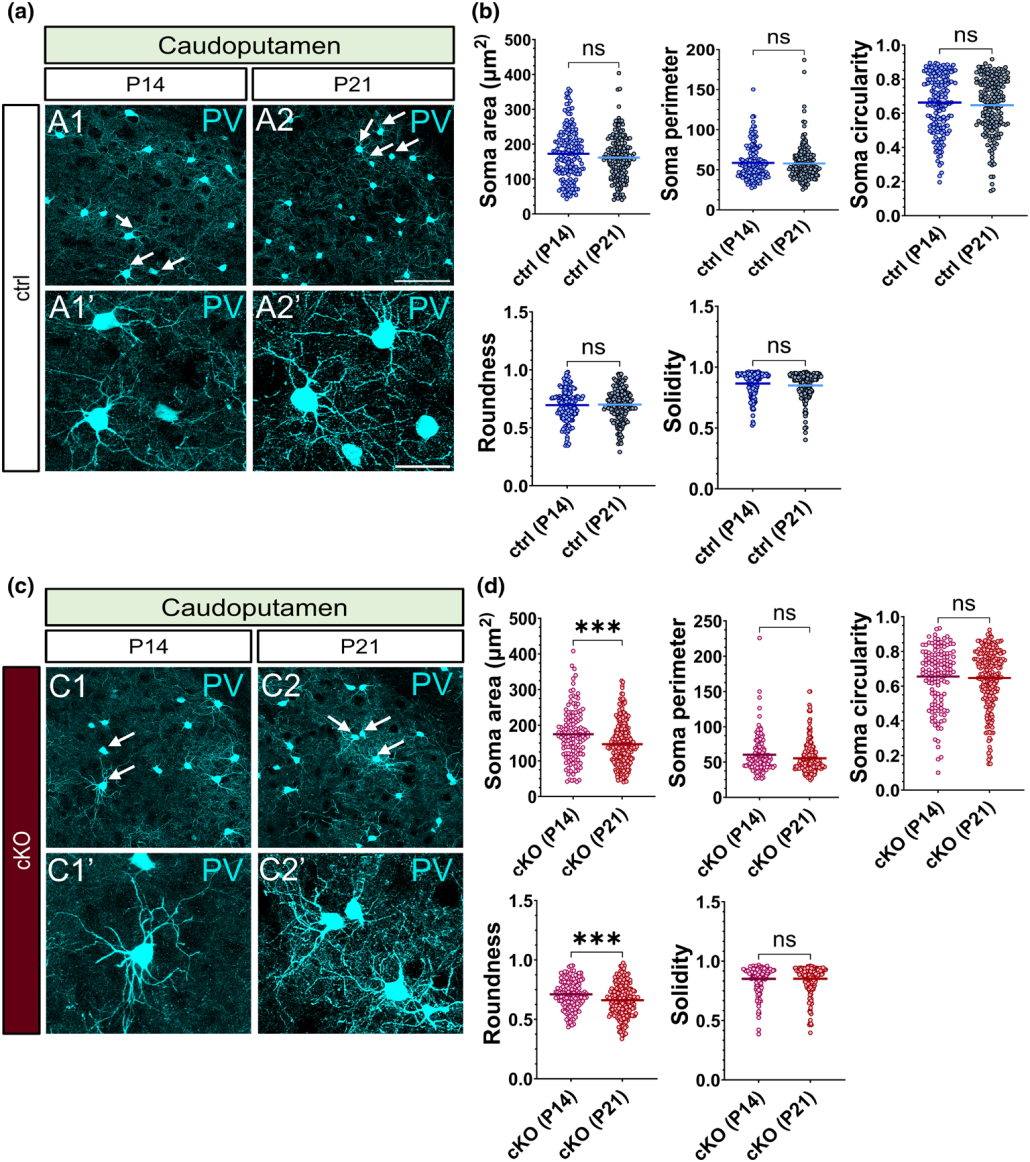
Striatal PV interneurons in the layer 5‐silenced brains (*Rbp4‐Cre*;*Ai14*; *Snap25* ^*fl/fl*^) display altered developmental dynamics of soma morphology between P14 and P21. Maximum intensity projected confocal z‐stack images of PV‐immunoreactive neurons in the caudoputamen in the control mice at P14 (A1, A1′) and P21 (A2, A2′). The dynamics of PV neuron morphology in the caudoputamen of control mice did not exhibit any significant alterations in the measured soma characteristics of PV+ neurons between P14 and P21 (b). Maximum intensity projected confocal z‐stack images of PV‐immunoreactive neurons in the caudoputamen in the *Snap25* cKO mice at P14 (C1, C1′) and P21 (C2, C2′). In the L5‐silenced animals, there was a considerable reduction in the soma area and soma solidity of striatal PV+ interneurons between P14 and P21 (d) (soma area, *p* = 0.0003, roundness, *p* = 0.0003. Unpaired *t*‐test with Welch's correction. P14, *n* = 136, P21, *n* = 236) ***p < 0.001. Arrows denote PV+ neurons shown in high‐magnification images. Scale bars: 100 μm (A2), 20 μm (A2′).

Having shown the chronic cessation of regulated vesicle release from L5 projection neurons alters the soma size and roundness of striatal PV+ neurons at P21, we next assessed the course of striatal PV+ morphology between P14 and P21 in the control brains. None of the examined characteristics of the morphology of PV neurons in the striatum showed significant alterations between P14 and P21 (Figure 6a,b). In the layer 5‐silenced brains; however, we revealed a significant reduction in the striatal PV+ neurons' soma area and roundness between 14 and P21 (Figure 6c,d). The somata of striatal PV+ cells in the *Snap25* cKO mice became more compressed, smaller and less rounded due to the considerable decrease in their soma area. Given that no such morphological changes were noted in the dynamics of PV+ neurons in the control striatum between P14 and P21, the altered morphology of striatal PV+ neurons in the cKO brains seemed to be caused by the chronic abolition of synaptic transmission from L5.

### 3.7 Chronic impairments in synaptic vesicle release from subsets of layer 5 projection neurons have no permanent impact on the morphology of interneurons in the adult striatum

**FIGURE 7:**
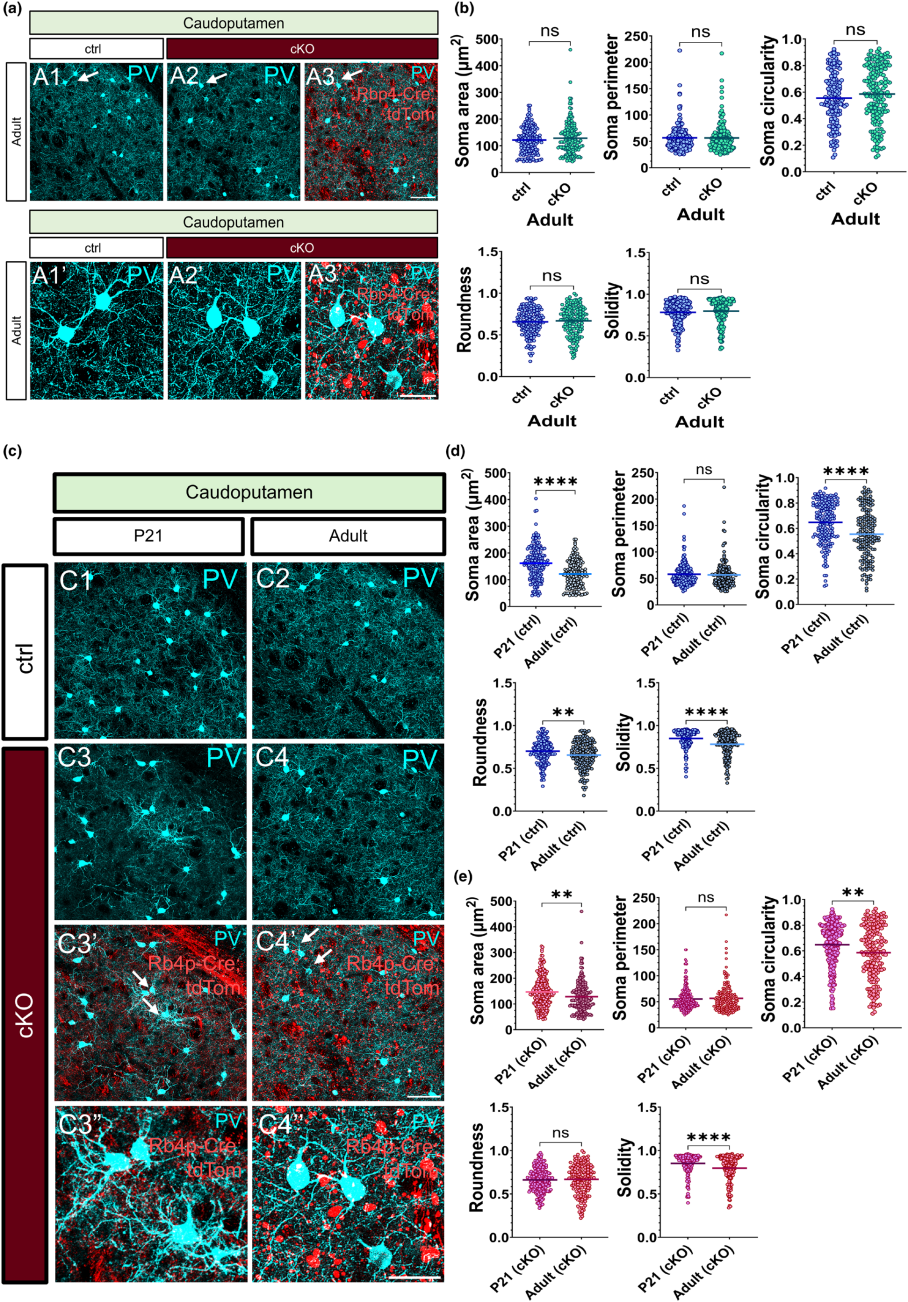
Chronic impairments in a subset of layer 5 projection neuron activity have no permanent impact on the soma morphology of interneurons in the adult striatum. Maximum intensity projected z‐stack images of PV+ interneurons in the caudoputamen in the control (A1, A1′) and the L5‐silenced mice at 3 months of age (A2, A2′). Superimposed maximum intensity projected z‐stack images of PV+ neurons (cyan) and *Rbp4‐Cre;Ai14;Snap25* ^*fl/fl*^ projection neurons (red) in the striatum of adult *Snap25* cKO mice (A3, A3′). White arrows mark the location of striatal PV+ neurons depicted in higher magnification images (A1′, A2′, A3′). No significant differences were observed in the soma parameters of PV+ neurons in the striatum of L5‐silenced mice compared to control mice at 12 weeks of age (b). PV+ interneurons in the caudoputamen of control mice at P21 (C1) versus 12 weeks of age (C2). PV+ interneurons in the caudoputamen of *Snap25* cKO mice at P21 (C3) and 12 weeks of age (C4). Axonal projections of *Rbp4‐Cre*;*Ai14*;*Snap25* ^*fl/fl*^ neurons (red) in the caudoputamen in the L5‐silenced mice at P21 (C3′, C3″) and 12 weeks of age (C4′, C4″). Note the stark contrast in the axonal fibres of L5 projection neurons between the developing vs. the adult *Snap25* cKO mice. Accumulation of tdTomato+ punctae and fragmentation of L5 axons were only observed in the adult *Snap25* cKO mice. Quantitative analysis of the dynamics of PV neuron morphology from P21 to 12 weeks of age disclosed a marked decrease in the soma area, circularity, roundness and solidity of striatal PV neurons in the control brains (d) (P21, *n* = 180; 12 weeks, *n* = 168 cells, **p < 0.01, ****p < 0.0001, unpaired *t*‐test with Welch's correction). Similar reductions were noted in the soma area, circularity and solidity of striatal PV+ neurons in the L5‐silenced mice between P21 and 12 weeks of age (e) (soma area, *p* = 0.0028, circularity, *p* = 0.0020, solidity, *p* = <0.0001) (P21, *n* = 236; 12 weeks, *n* = 171 cells). Scale bars: 50 μm (A3, C4′), 20 μm (A3′, C4″).

To further investigate if the perceived changes in the morphology of PV interneurons at P21 persist into adulthood, we conducted the same morphometric analyses on striatal PV neurons in the adult brain. Interestingly, none of the observed changes in PV morphology were present at 3 months of age in the L5‐silenced brains (Figure 7a,b). This suggests that L5 causes temporary changes in the morphology of striatal PV neurons. Next, we assessed how the dynamics of PV morphology changed from development to adulthood. The striatal PV neurons in the control brains exhibited distinct alterations in soma area, circularity, roundness and solidity. By 3 months of age, all these characteristics had significantly decreased (Figure 7c,d). Similar decreases in soma area, circularity and solidity were observed in the *Snap25* cKO brains (Figure 7c,e). These results demonstrate that chronic silencing of L5 activity modifies the morphological dynamics of striatal PV neurons as well as their soma characteristics at P21 while leaving their number intact. It is tempting to speculate that the chronic modulation of Ca^2+^‐dependent synaptic transmission in L5 has a transient effect on striatal PV+ neurons, given that the observed phenotype was rescued by 12 weeks of age. Please refer to Table S4 for an overview of the morphometrics data.

### 3.8 The absence of regulated vesicle release from Rbp4‐Cre + projection neurons does not alter the laminar distribution of PV interneurons in the juvenile cortex

**FIGURE 8:**
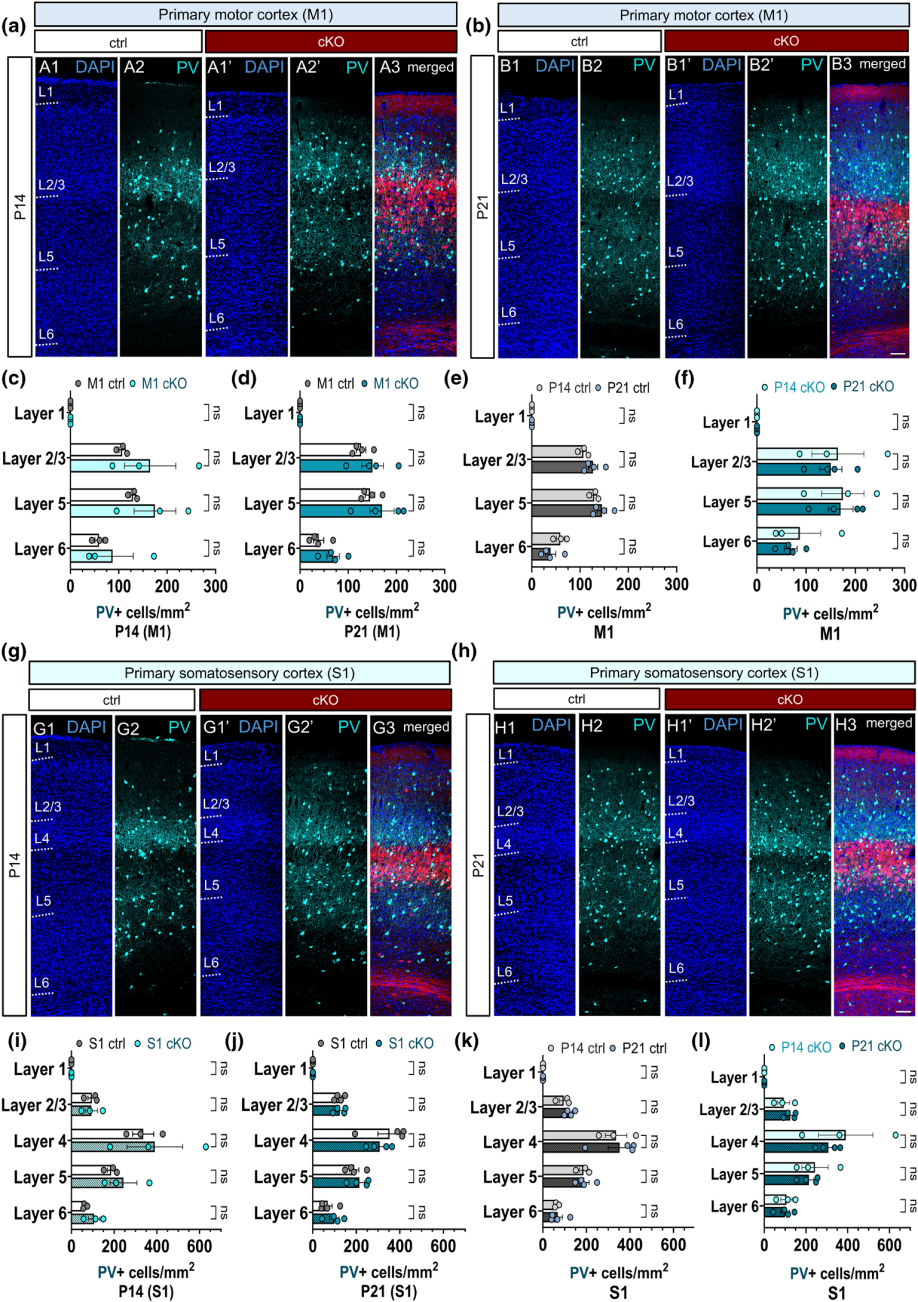
Chronic abolition of Ca^2+^−dependent neurotransmission from a subset of layer 5 pyramidal neurons does not reorganise the laminar distribution of PV interneurons in the juvenile cortex. Laser‐scanning confocal tile‐scan images of PV+ immunostaining in the L5‐silenced control and cKO brains at P14 and P21. Nuclear counterstaining using DAPI was performed to define the boundaries of cortical layers in M1 (A1, A1′, B1, B1′) and S1 (G1, G1′, H1, H1′). Confocal tile scans showing the distribution of PV+ neurons across the cortical layers in M1 at P14 (A2, A2′) and P21 (B2, B2′). Merged confocal images displaying *Rbp4‐Cre*;*Ai14*;*Snap25* ^*fl/fl*^ projection neurons in red and PV‐immunoreactive neurons in cyan in M1 (A3, B3) and S1 (G3, H3). Quantification of the laminar distribution of PV+ neurons in M1 at P14 and P21 (c, d). No significant differences were found in the density of PV neurons in any cortical layers in M1 between the control and the L5‐silenced brains at P14 and P21 (c, d). The developmental dynamics of the distribution of PV neurons in M1 in the L5‐silenced control brains did not reveal significant alterations in any cortical layers (e). The dynamics of the laminar distribution of PV neurons in M1 remained unaltered in the chronically silenced L5 brains between P14 and P21 (f). Confocal tile scans depicting the distribution of PV+ neurons across the cortical layers in S1 at P14 (G2, G2′) and P21 (H2, H2′). The distribution of PV neurons across the cortical layers in S1 showed no differences between P14 and P21 in the control and the L5‐silenced brains (g–j). No differences were noted in the dynamics of the laminar distribution of PV+ neurons between P14 and P21 in the S1 control and the L5‐silenced brains (k, l). Scale bars: 100 μm. All data is represented as mean ± SEM values. 2‐way ANOVA with Šídák's multiple comparisons test. M1: Primary motor cortex, S1: Primary somatosensory cortex.

After establishing that the cessation of neurotransmitter release from L5 projection neurons does not affect the density of maturing PV interneurons, we next investigated whether it altered their laminar distribution. No differences were detected in the laminar positioning of PV in M1 at P14 (Figure 8a,c A1–A2′) at P21 (Figure 8b,d B1–B2′). We found that the absence of regulated vesicle release from L5 projection neurons did not alter the distribution of PV in any cortical layers in S1 at P14 either (Figure 8g,i G1‐G1′). It can be concluded that Ca^2+^−dependent neurotransmission from layer 5 does not regulate the spatial organisation of PV neurons during postnatal development (Figure 8h,j H1‐H1′).

We next sought to examine if developmental age affected the spatial organisation of PV. Between P14 and P21, we did not observe a significant change in the laminar distribution of PV neurons in the control brains in M1 (Figure 8e). The developmental dynamics of PV neurons in M1 in the L5‐silenced brains were identical to that of the control brain (Figure 8f). Regarding the dynamics of PV distribution in S1 between P14 and P21, we observed no alterations in their laminar positioning in S1 in any cortical layers in the control brains (Figure 8k). Silencing subsets of layer 5 pyramidal neurons had no impact on the laminar distribution of PV in S1 either (Figure 8l). These results indicate that the chronic manipulation of the activity of L5 neurons leaves the dynamics of PV distribution intact and does not rearrange the spatial organisation of PV during development. In agreement with the density results, we did not observe significant changes in the laminar density of PV neurons either in M1 or S1 between P14 and P21.

### 3.9 Chronic cessation of evoked vesicle release from L5 projection neurons alters the correlation between PV interneurons and the perineuronal nets in the adult motor cortex

**FIGURE 9:**
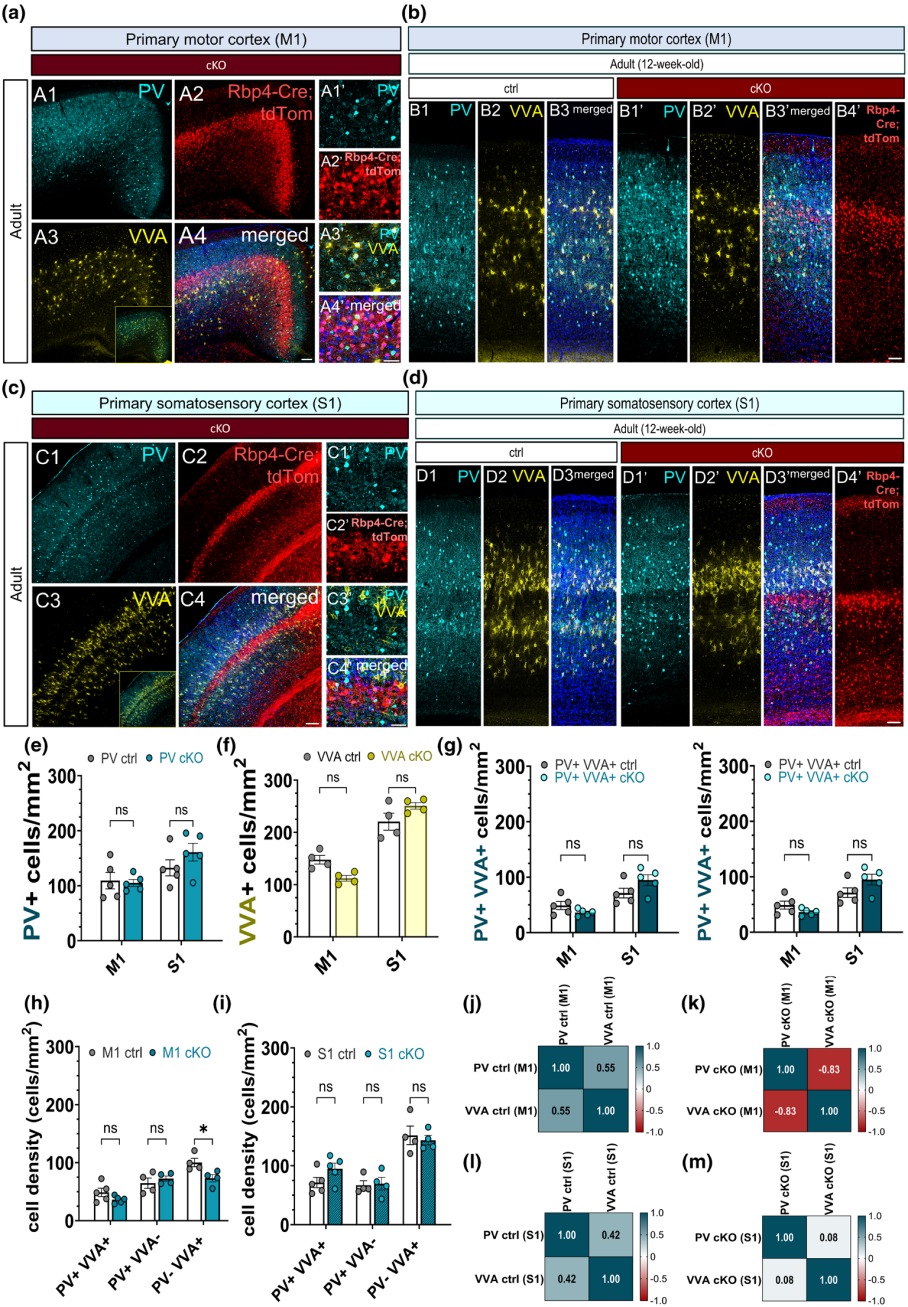
Chronic abolition of layer 5 pyramidal neuron activity reverses the correlation between PV interneurons and the perineuronal nets in M1, but it has no long‐term effect on the density of PV neurons in the adult cortex. Low‐magnification confocal images of the primary motor cortex in the *Snap25* cKO brains at 3 months of age (a). PV‐immunoreactive neurons are shown in cyan (A1, A1′), *Rbp4‐Cre*;*Ai14*;*Snap25* ^*fl/fl*^ projection neurons in L5 of M1 are seen in red (A2, A2′). The cells that are labelled with *Vicia villosa* agglutinin demonstrate the presence of perineuronal nets encapsulating PV interneurons (A3, A3′). Merged confocal images show colocalisation of PV and VVA in M1 in *Snap25* cKO brains that are highly concentrated near the cell bodies of L5 *Rbp4‐Cre*;*Ai14*;*Snap25* ^*fl/fl*^ projection neurons (A4, A4′). Maximum intensity projected z‐stack and tile‐scan images of M1 depicting PV‐immunostained neurons (B1, B1′) and VVA‐positive PNN labelling in the control (B2) and L5‐silenced brains at 12 weeks of age (B2′, B3). *Rbp4‐Cre*;*Ai14*;*Snap25* ^*fl/fl*^ projection neurons in the adult motor cortex showing signs of degeneration and axonal fragmentation (B4′). Single‐scan confocal images of PV+ neurons (C1, C1′) and VVA+ cells in the primary somatosensory cortex of Snap25 cKO brains at 3 months of age (C3, C3′). The degeneration of Rbp4‐Cre + neurons in layer 5 is a clear indication of the ongoing degenerative processes in *Snap25* cKO mice (C2). Note the dying *Rbp4‐Cre*;*Ai14*;*Snap25* ^*fl/fl*^ neurons at 3 months of age (C2′). PV+ and VVA+ signals superimposed on the *Rbp4‐Cre*,*Ai14*;*Snap25* ^*fl/fl*^ neurons (C4, C4′). Tile‐scan and maximum intensity projected z‐stack confocal images of PV‐immunoreactive neurons (D1, D1′) and VVA‐labelled PNNs in the control and L5‐silenced brains at 12 weeks of age (D2, D3, D2′, D3′). Degenerating *Snap25*‐deleted Rbp4‐Cre neurons in S1 in the adult *Snap25* cKO mice. Note the accumulation of tdTomato‐positive punctae throughout the cortex (D4′). Quantification of PV+ and VVA+ cells in M1 and S1 at 3 months of age revealed no significant differences in the density of PV+ and VVA+ neurons between the control and the L5‐silenced mice (e, f). The percentage of PV+ VVA+ cells in M1 was significantly lower in the *Snap25* cKO mice at 3 months of age (*p* = 0.0081, 2‐way ANOVA with Šídák's multiple comparisons test (g). There was a significant decrease in the density of PV‐ VVA+ neurons in the adult motor cortex in the *Snap25* cKO mice (h) (*p* = 0.0263, 2‐way ANOVA with Šídák's multiple comparisons test). Different subsets of PV neurons were not altered significantly in the adult S1 between the control and the *Snap25* cKO mice (i). PV‐VVA correlation was reversed in M1 as the Snap25 cKO mice exhibited a negative correlation between PV and VVA (j, k) (*r* = −0.828). PV‐VVA correlation in S1 in the *Snap25* cKO mice was not altered and followed the positive correlation seen in control mice (l, m). *p < 0.05, **p < 0.01. Scale bars: 100 μm (A4, C4), 50 μM (A4′, C4′, B3, D4).

We next explored whether chronically silencing L5 permanently impacted PV interneurons. To investigate whether different subsets of PV interneurons were differentially affected, we visualised the perineuronal nets (PNNs) using the *Vicia villosa* agglutinin (VVA) that allowed us to differentiate between PV neurons with PNNs (PV+ VVA+) and PV neurons without PNNs (PV+ VVA‐). At 3 months of age, no differences were observed in the density of PV cells in M1 and S1 between the control and the cKO brains even though the *Rbp4*‐*Cre*;*Snap25* ^*fl/fl*^ L5 neurons display clear signs of neurodegeneration and neuroinflammation by this age (Figure 9a A2, A2′, Figure 9b B4′, Figure 9c C2, C2′, Figure 9d D4′) (see Hoerder‐Suabedissen et al., 2019). Considering the drastic impairments of the silenced L5 projections, it is intriguing that cortical PV interneurons in M1 and S1 remain unaffected by the ongoing neurodegenerative events in the adult *Snap25* cKO mice (Figure 9a A1, A1′, Figure 9b B1, B1′, Figure 9c C1, C1′, Figure 9d D1, D1′, Figure 9e). To determine whether silencing L5 alters the PNNs, we assessed the density of VVA+ cells in the adult cortex. There were no changes in the density of VVA+ cells in M1 and S1 between the control and the cKO brains (Figure 9a A3, Figure 9b B2, B2′, Figure 9c C3, Figure 9d D2, D2′, Figure 9f).

After confirming the abolition of evoked vesicle release had no long‐term impact on the density of PV interneurons and the PNNs, we sought to explore changes in the subtypes of cortical PV interneurons. Three groups were distinguished based on the presence of PNN: (1) PV neuron with PNNs or the double positives (PV+ VVA+); (2) PV neuron without PNNs (PV+ VVA‐); (3) PNNs around non‐PV neurons (PV‐ VVA+). While the density of the three subgroups (PV+ VVA+, PV+ VVA‐, PV‐ VVA+) remained unchanged in S1 in the L5‐silenced adult brains when compared to the control brains (Figure 9i); there was a significant decline in the density of the PV‐ VVA+ cells in M1 in the *Snap25* cKO brains (Figure 9h). Notably, the decrease in the density of PV‐ VVA+ cells is happening without a change in the density of PV+ neurons, indicating that the PNN is still plastic and capable of dynamic changes in the adult brain. We also noted a significant decrease in the percentage of the double‐positive cells (PV+ VVA+) in M1 in the layer 5‐silenced mice (Figure 9g). Next, we explored how the chronic abolition of regulated vesicle release from L5 influenced the correlation between PV and VVA in different cortical regions. The PNN is thought to be predominantly surrounding PV interneurons, therefore the positive association between PV and VVA in the control M1 and S1 areas is not an unanticipated result (Figure 9j,l). However, this correlation was reversed in the motor cortex of L5‐silenced mice (Figure 9k). It is noteworthy that the effect of L5 appears to vary depending on the cortical region as, although decreasing, the correlation between PV and VVA in S1 did not revert (Figure 9m). The differential impact on M1 and S1 points to a variable degree of plasticity and correlation between PV and VVA in the adult cortices. For an overview of the adult and developmental PV and VVA results, please refer to Table S5.

### 3.10 Chronic silencing of subsets of layer 5 projection neurons differentially alters the laminar positioning of PV interneurons and the perineuronal nets in the adult motor and sensory cortices

**FIGURE 10:**
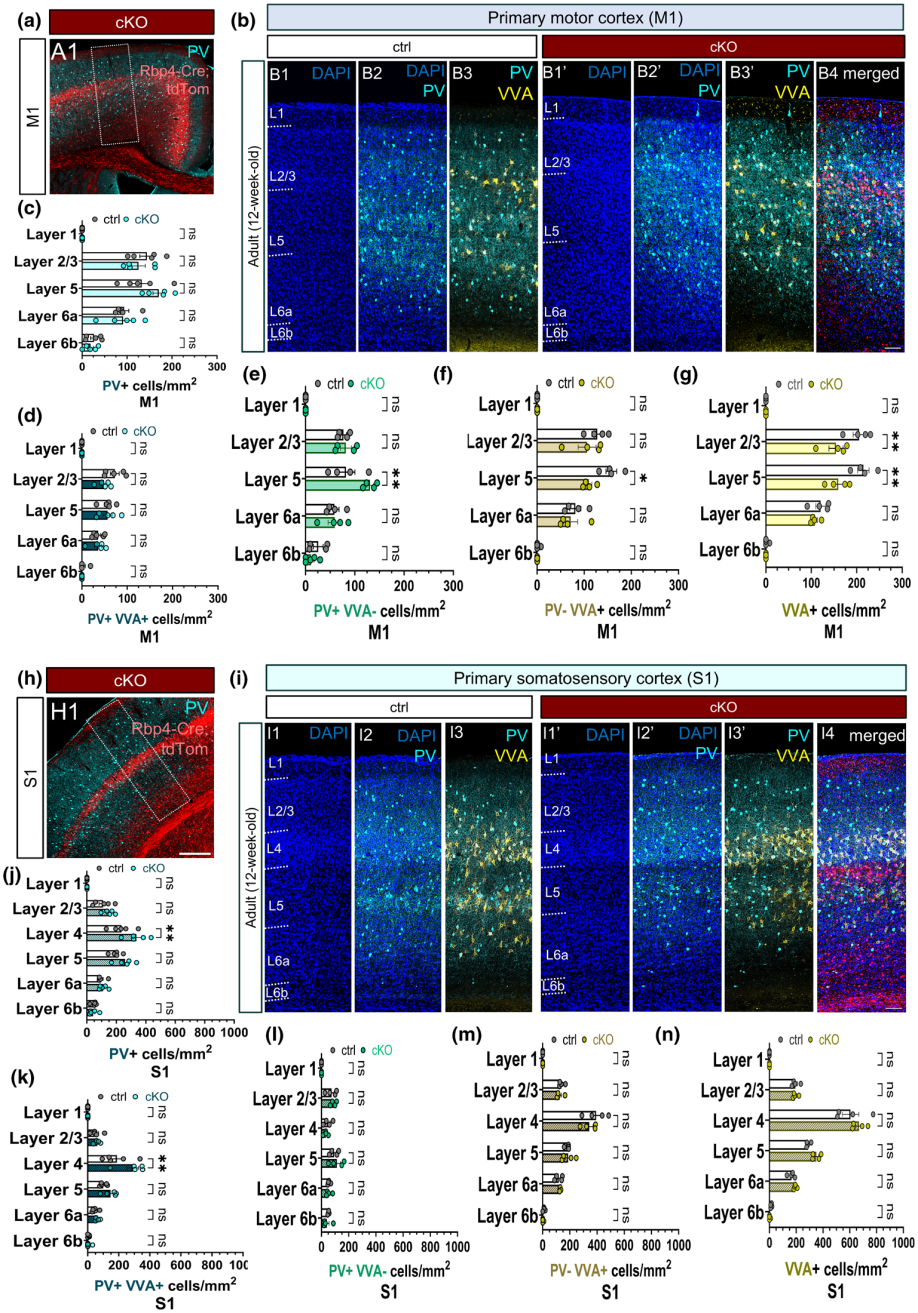
Chronic silencing of a subpopulation of layer 5 projection neurons rearranges and differentially impacts the laminar distribution of PV interneurons and the perineuronal nets in the adult primary motor and somatosensory cortex. Low‐magnification point‐scanning confocal image of the primary motor cortex (a) at 3 months of age illustrating PV interneurons (cyan) and L5‐silenced projection neurons (red) (A1). The imaging site is outlined by a dotted white rectangular. Combined tile‐scan and maximum intensity projected z‐stack images representing the laminar distribution of PV+ and VVA+ cells in the control and the *Snap25* cKO mice in M1 (B1–B4). White dotted lines drawn to delineate cortical layer boundaries. Laminar distribution analysis showed no significant changes in the density of PV+ and PV+ VVA+ in M1 at 12 weeks of age between the adult control and the L5‐silenced brains (c, d). However, the *Snap25* cKO motor cortex demonstrated a considerable rise in the density of PV+ VVA cells in L5 (e). The density of VVA+ cells was significantly reduced in the *Snap25* cKO mice both in L2/3 and L5 in M1 (f, g) (L2/3, *p* = 0.0028; L5, *p* = 0.0011, 2‐way ANOVA with Šídák's multiple comparisons test). Low‐magnification point‐scanning confocal image of the primary somatosensory cortex (h) at 3 months of age illustrating PV interneurons (cyan) and L5‐silenced projection neurons (red) (H1). Combined tile‐scan and collated z‐stack images showing the laminar distribution of PV+ and VVA+ cells in the control and the *Snap25* cKO mice in S1 (I1–I4). Laminar distribution analysis of S1 revealed a significant rise in the density of PV+ neurons in L4 at 3 months of age in the L5‐silenced mice (j) (*p* = 0.0063). The density of double‐positive PV+ VVA+ neurons followed the same trend in L4 in S1 (l) (*p* = 0.0023). The laminar distribution of VVA+, PV+ VVA‐ and PV‐ VVA+ neurons was not altered significantly in any cortical layers in S1 at 3 months of age (k, m, n). Statistical tests: 2‐way ANOVA with Šídák's multiple comparisons. Scale bars: 200 μm (H1), 50 μm (B4, I4). M1: Primary motor cortex, S1: Primary somatosensory cortex, PV, Parvalbumin; VVA, *Vicia villosa* agglutinin.

We next sought to examine the laminar distribution of PV neurons and the PNNs in the adult primary motor and somatosensory cortices. The chronic cessation of evoked vesicle release from L5 that leads to the degeneration of L5 neurons, and the disintegration of its long‐range axonal projections may have permanent effects on the GABAergic interneurons that might not be apparent in the postnatal brain. At 3 months of age, we found a significant reduction in the density of the PNN in L5 and L2/3 of the motor cortex in the L5‐silenced mice (Figure 10d) and a similar trend was noted for L5 PV‐ VVA+ cells (Figure 10g). Of the subpopulations of PV neurons, only the PV+ VVA‐ subgroup showed a significant increase in L5 in the M1 region of the *Snap25* cKO brains. This finding indicates that PV+ neurons without PNNs may re‐enter a plastic phase by retaining their capacity to dynamically respond to changes in network activity even after the consolidation of inhibitory networks and the closure of critical windows (Figure 10a–c,e,f). In contrast to M1, no differences were detected in the laminar distribution of VVA+ and PV‐VVA+ cells in S1 in the L5‐silenced brains (Figure 10k,n). However, both the density of PV+ and PV+ VVA+ neurons rose significantly in L4 of S1 in the *Snap25* cKO brain (Figure 10j,l).

Considering that the chronic abolition of synaptic vesicle release from L5 projection neurons has altered the laminar distribution of PV+ neurons and the PNNs only in the adult cortex, it is tempting to conclude that the effect of chronic silencing of L5 on PV neurons is long‐term and not manifested until adult stages. Since the total density of PV and VVA cells remained unchanged, the chronic silencing of L5 does not lead to interneuron cell death and/or loss of PNNs in the adult brain.

### 3.11 The correlation between PV and the perineuronal net varies by cortical layers

**FIGURE 11:**
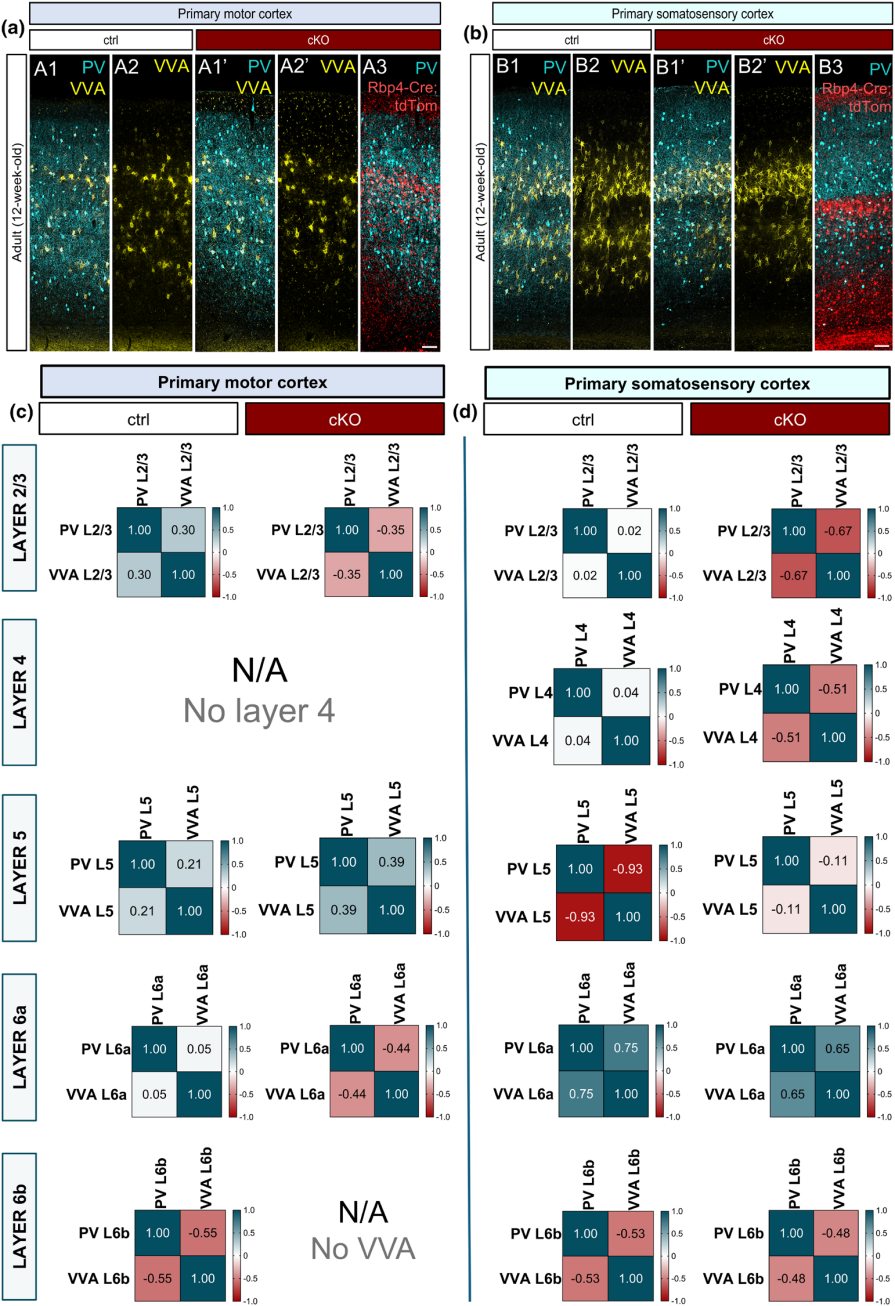
Chronic silencing of the activity of a subset of layer 5 projection neurons unveils regional and layer‐dependent differences in the correlation between cortical PV interneurons and the perineuronal nets. Combined tile‐scan and maximum intensity projected z‐stack confocal images depicting PV‐immunostained neurons, VVA‐labelled PNNs and the degenerating *Rbp4‐Cre*;*Ai14*;*Snap25* ^*fl/fl*^ projection neurons in M1 and S1 in the control (A1, A2, B1, B2) and the chronically silenced L5 brains at 12 weeks of age (A1′–A3, B1′–B3). Heatmaps illustrating the Pearson correlation between PV+ and VVA+ cells in all cortical layers in M1 at 12 weeks of age (c). Note that the superficial and the infragranular cortical layers in M1 exhibit varying levels of correlation between PV+ and VVA+ in the control and the *Snap25* cKO brains. In the *Snap25* cKO brains, there were no VVA+ cells in L6b in M1. The direction of correlation between PV+ and VVA+ was reversed in the L5‐silenced mice in L2/3 and L6a of M1. Heatmaps illustrating the Pearson correlation between PV+ and VVA+ cells in all cortical layers in S1 at 12 weeks of age (d). Similar to M1, the correlation between PV and VVA in S1 varies greatly across cortical layers. Note that both the control and the *Snap25* cKO mice show a negative correlation in L6b between PV and VVA at 12 weeks of age. Numbers in heatmaps represent *r* values of Pearson correlation. Scale bars: 50 μm (A3, B3). M1: Primary motor cortex, S1: Primary somatosensory cortex.

We previously established that the degree of correlation between PV and VVA neurons varied between different cortical regions and the chronic abolition of regulated vesicle release from subsets of layer 5 projection neurons disrupted the positive PV‐VVA correlation in the adult primary motor cortex in the *Snap25* cKO brains (Figure 8j,k). Considering that the correlation between PV and VVA can also be influenced by the laminar position of PV neurons and PNNs, we next examined the Pearson correlation between PV+ and VVA+ immunoreactive cells in different cortical layers of M1 and S1 at 12 weeks of age. Specifically, whether the association between PV and VVA cell density varied between the upper and lower cortical layers. In M1, the *Snap25* cKO brains displayed a negative correlation between PV and VVA in L2/3 and L6a, in contrast to the control brains' positive correlation in L2/3 and L6a (Figure 11c). In the L5‐silenced brains, the correlation between PV and VVA in L2/3 and L4 of S1 was reversed (Figure 11d).

The negative correlation between PV and VVA in the *Snap25* cKO mice indicates that not all PV+ cells are surrounded by PNNs and while most PV+ neurons are VVA+ in L4 of S1, there are PNNs around neurons that are PV‐. Although the correlation remained unaltered in L6a and L6b in the *Snap25* cKO brains, the negative correlation in L6b observed both in the control and the cKO brains supports our density data showing that VVA rarely occurs in L6b. The findings imply that the correlation between PV and VVA changes in degree throughout cortical layers and between the motor and somatosensory cortices. These results also demonstrate that upper and lower cortical layers in functionally distinct cortical areas have different correlation values. Establishing that PV‐VVA correlation is both region‐ and layer‐dependent further highlights how dynamically PV and PNNs interact, and how their link is more nuanced than previously believed.

### 3.12 Persistently silencing subsets of layer 5 projection neurons does not lead to long‐term changes in the number of subcortical PV neurons in the projection regions of layer 5

**FIGURE 12:**
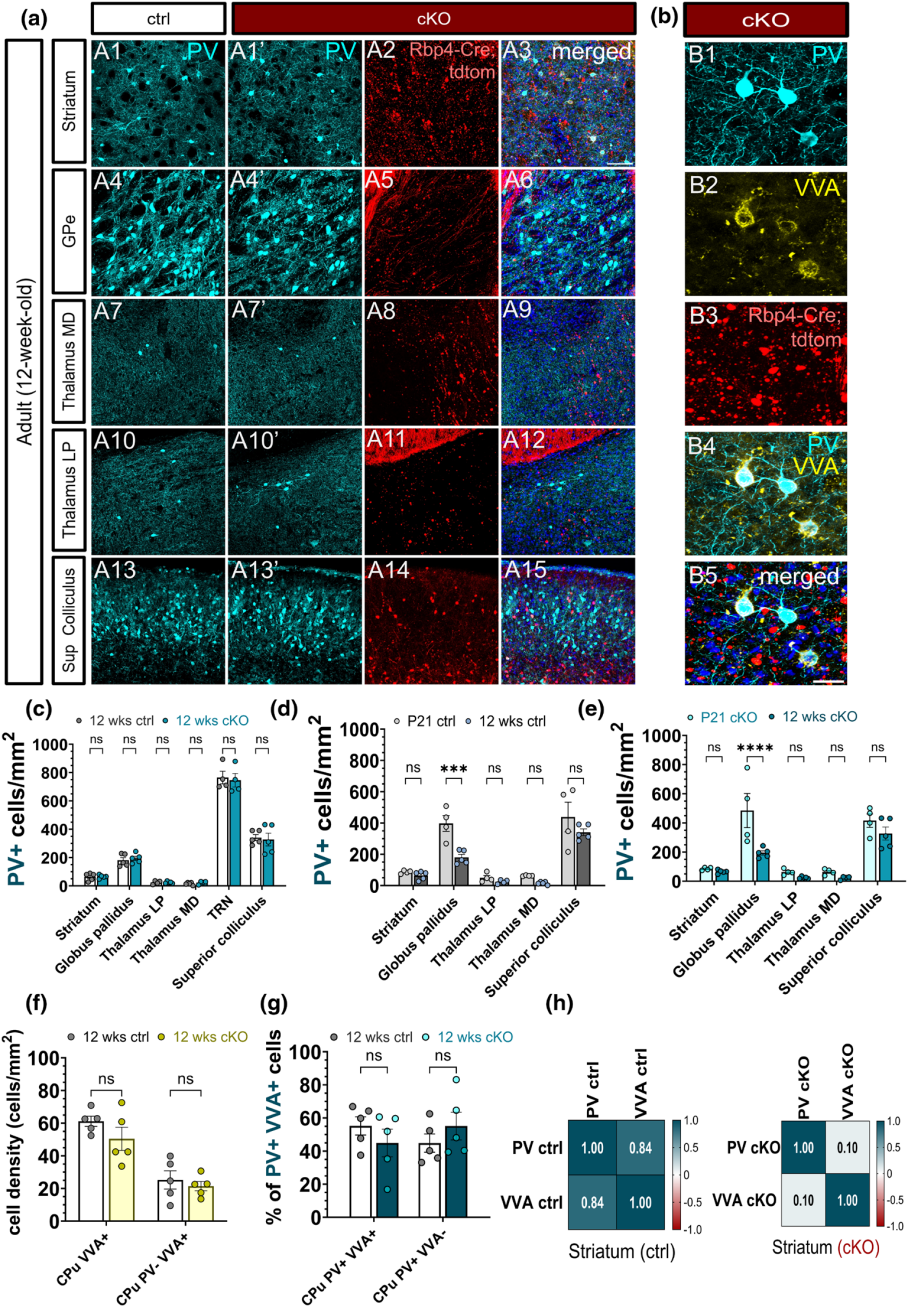
Loss of synaptic vesicle release from Rbp4‐Cre + layer 5 projection neurons has no long‐term impact on the density of PV interneurons in the subcortical projection regions. Maximum intensity projected confocal z‐stack images demonstrating the selected output regions of the silenced Rbp4‐Cre projection neurons at 3 months of age. Subcortical PV+ neurons in the output regions of L5 are shown in cyan, while the *Rbp4‐Cre*;*Ai14*;*Snap25* ^*fl/fl*^ are depicted in red. PV‐immunopositive neurons in the caudoputamen and the external segment of the globus pallidus in the control (A1, A4) and the *Snap25* cKO mice (A1′, A4′). PV+ neurons in the higher‐order lateral posterior and mediodorsal thalamic nuclei in the control (A7, A10) and the *Snap25* cKO mice (A7′, A10′). PV‐immunoreactive neurons in the superior colliculus at 12 weeks of age in the control (A13) and the L5‐silenced mice (A13′). Long‐range corticofugal projections of *Rbp4‐Cre*;*Ai14*;*Snap25* ^*fl/fl*^ cortical pyramidal neurons in the adult Snap25 cKO mice in the basal ganglia (A2–A6), the higher‐order thalamic nuclei (A8–A12) and the midbrain (A14–A15). Note the disintegration of the projections and the enlarged tdTomato+ swellings on the axonal fragments of *Rbp4‐Cre*;*Ai14*;*Snap25* ^*fl/fl*^ neurons. Point‐scanning maximum intensity projected z‐stack images of striatal PV+ neurons and VVA‐labelled PNNs at 12 weeks of age in the *Snap25* cKO mice (B1, B2). Disintegrating axonal fibres of the silenced L5 projection neurons in the caudoputamen (B3). Presence of perineuronal nets (PNNs) surrounding striatal PV+ neurons in adult L5‐silenced mice (B4, B5). Quantification of PV+ neurons in the output regions of *Rbp4‐Cre*;*Ai14*;*Snap25* ^*fl/fl*^ neurons at 12 weeks of age. No subcortical regions showed statistically significant variations in PV+ neuron density between the *Snap25* cKO and control groups (c). Both in the control and the *Snap25* cKO mice, the dynamics of PV+ neurons between P21 and 3 months of age showed a marked decrease in PV+ neuron density in the globus pallidus (****p < 0.0001, 2‐way ANOVA with Šídák's multiple comparisons test) (d, e). There were no significant variations in the density of VVA+ and PV‐ VVA+ cells in the adult striatum (f). Bar graphs representing the percentage of PV+ VVA+ and PV+ VVA‐ cells in the caudoputamen between control and *Snap25* cKO mice at 12 weeks of age (g). In the striatum, the layer 5‐silenced animals (h) exhibited a reduction in the degree of positive correlation between PV and VVA (h). Scale bars: 50 μm.

To investigate the long‐term influence of the chronic cessation of evoked vesicle release on PV interneurons, we analysed the density of subcortical PV neurons in the output regions of L5 neurons at 3 months of age. It is important to note that at this age, most subcortical axons from the silenced L5 projection neurons have degenerated. Surprisingly, the layer 5‐silenced brains did not reveal significant alterations in the density of subcortical PV neurons in any of the long‐range projection regions of L5 (Figure 12a,c). In the striatum, neither the density of the PNN nor the density of PV‐ VVA+ cells changed in the adult *Snap25* cKO brains (Figure 12b,f,g). To determine if chronically abolishing neurotransmitter release from L5 changes the maturation of PV neurons into adulthood, we next looked at the dynamics of subcortical PV neurons from development to adulthood. First, we examined the course of maturation of subcortical PV neurons between P21 and 3 months of age in the control brains where we noted a significant decrease in PV cell density in the GPe (Figure 12d). We noticed the same trend in layer 5‐silenced brains. Of the output regions of L5, the density of PV neurons was only altered in the GPe, while the rest of the projection sites and the cortical regions remained unchanged (Figure 12e). These findings are consistent with the idea that the first week of development marks the most significant milestones in the maturation of PV neurons, and when these neurons undergo developmental apoptosis, their populations appear to stabilise and hold steady into adulthood (Denaxa et al., 2018; Magno et al., 2021; Southwell et al., 2012; Wong et al., 2018, 2022). We next examined whether PV density correlated with VVA density in subcortical regions. Although the striatum of the layer 5‐silenced mice showed a positive PV‐VVA correlation (Figure 12h), the level of correlation was not as high as that of the control brains. These results suggest that the chronic abolition of Ca^2+^‐dependent neurotransmission from L5 neurons has a differential impact on cortical and subcortical brain regions, and the correlation between PV and VVA may be region‐specific.

## 4 DISCUSSION

We explored the role of deep‐layer projection neurons in the spatial and laminar organisation of cortical and subcortical parvalbumin neurons at distinct developmental stages and in adulthood. Using a triple transgenic conditional knockout mouse (*Rbp4*‐*Cre*;*Ai14*;*Snap25* ^*fl/fl*^), we selectively ablated *Snap25* from Rbp4‐Cre‐expressing neurons that abolished evoked synaptic vesicle release from subsets of layer 5 corticostriatal pyramidal neurons (Gustus et al., 2018; Hayashi et al., 2021; Hoerder‐Suabedissen et al., 2019; Korrell et al., 2019; Krone et al., 2021; Marques‐Smith et al., 2016; Welch et al., 2000). We tested the hypothesis that infragranular pyramidal cells instruct the organisation of inhibitory cortical circuits by fine‐tuning the number of GABAergic cells via activity‐dependent mechanisms. We found that perturbing the function of infragranular pyramidal cells by abolishing Ca^2+^‐dependent neurotransmission from L5 did not impede the development of PV+ neurons; however, it rearranged the laminar distribution of PV+ cells in the adult motor and somatosensory cortex. We demonstrated that chronic silencing of L5 had a differential impact on PV neurons and the PNNs by affecting different cortical layers. While the absence of vesicle release from L5 impacted PV+ neurons in L4 in S1, it altered the laminar distribution of VVA+ and PV‐ VVA+ neurons in L2/3 and L5 in M1. These results demonstrate that different cortical layers and distinct subtypes of PV+ neurons respond differently to the abolition of vesicle release from L5 projection neurons. We also discovered that the correlation between PV and VVA varies by cortical regions and layers and such correlation can be disrupted in the adult brain by chronically manipulating L5 projection neurons.

Recent research has demonstrated that the bidirectional manipulation of pyramidal neuron activity using Designer Receptors Exclusively Activated by Designer Drugs (DREADDs) only elicited a change in the density of PV+ and SST+ interneurons if the chemogenetic manipulation was performed between P5 and P8 (Wong et al., 2018, 2022). Considering that no such effect was detected when the pyramidal cell activation/inhibition occurred after the developmental apoptosis of GABAergic cells between P10 and P13, it appears there is a critical window of development when the pyramidal neuron activity is indispensable for the survival of GABAergic neurons. Developmental interneuron apoptosis is reported to occur between P5 and P10 with a peak at P7 (Lim et al., 2018; Pfisterer & Khodosevich, 2017; Southwell et al., 2012; Wong et al., 2018); therefore, most studies restricted the manipulation of pyramidal neuron activity to the window of programmed cell death (Sreenivasan et al., 2022; Wong et al., 2018, 2022) or carried out embryonic and/or neonatal interventions (Denaxa et al., 2018; Duan et al., 2020; Priya et al., 2018). In our study, pyramidal cell activity is irreversibly and chronically silenced in the *Rbp4‐Cre*;*Ai14*;*Snap25* ^*fl/fl*^ mice and thus, our results cannot directly be compared to the results of studies using transient modulation of pyramidal cell activity (*days* vs. *lifelong* manipulation). However, the mixed outcomes of pyramidal neuron activity on interneuron density reveal that GABAergic cells have different degrees of vulnerability during development highlighting the importance of studying GABAergic neurons at distinct stages of development.

Previous research has highlighted the role of cortical control in the regulation of striatal PV+ interneurons in the developing brain and argued that the cortical inputs determine the ultimate number of interneurons (Sreenivasan et al., 2022). Our study has different findings since the density of PV+ immunoreactive neurons in the striatum remained unchanged at P14 and P21 following the conditional deletion of *Snap25* from L5 projection neurons. While we detected changes in the morphology of striatal PV interneurons at P21; however, these changes diminished in the adult brain suggesting that the cessation of neurotransmitter release from L5 only had a transient effect on PV morphology. Surprisingly, the developmental dynamics of PV morphology was altered in the layer 5‐silenced mice.

The differences in findings regarding the density of striatal PV neurons may be explained by variations in the methods used to manipulate pyramidal neuron activity and the temporal differences in these manipulations. Sreenivasan and colleagues performed DREADD injections to carry out an acute and reversible manipulation of pyramidal neuron activity for days. As opposed to our research, which sought to detect long‐term alterations in the distribution of PV neurons by employing a chronic and irreversible modification of L5 pyramidal neurons. Another interesting question related to the role of corticostriatal pyramidal neurons in establishing the final number of interneurons both in cortical and subcortical circuits is the conditional deletion of different SNARE proteins from L5 projection neurons. Our results showed that the genetic ablation of *Snap25* from subpopulations of Rbp4‐Cre‐expressing L5 projection neurons left the density and laminar distribution of PV interneurons intact during the early stages of development both in the cortex and the subcortical innervation sites of L5. This contradicts the results of earlier studies where another fusion protein of the SNARE machinery, that is syntaxin‐binding protein 1 (*Syt1*), was genetically ablated from Rbp4‐Cre + neurons and caused a reduction in the number of PV neurons in the striatum (Sreenivasan et al., 2022).

The question arises as to whether the loss of different presynaptic SNARE fusion proteins has a differential impact on PV cell number. Our results suggest that Snap25 and Ca^2+^‐dependent synaptic transmission is dispensable for PV survival given the lack of local and global effect of abolished vesicle release from subgroups of cortical L5 projection neurons. However, in agreement with other studies (Sreenivasan et al., 2022), the conditional ablation of *Munc18*‐*1* from Rbp4‐Cre + L5 neurons only had a subtle effect on striatal PV density. This is an interesting observation considering that the removal of *Munc18*‐*1* results in the total cessation of neurotransmitter secretion (both *evoked* and *spontaneous* vesicle release are abolished) (Verhage et al., 2000). The ablation of *Snap25* in our mouse model only affected regulated synaptic exocytosis and it is tempting to speculate that more severe perturbations in synaptic transmission would lead to a more severe phenotype. However, one probable explanation for the lack of drastic changes in the density and distribution of PV interneurons might be that the deletion of the presynaptic SNARE proteins only affects a portion of L5 pyramidal neurons. We have previously documented that 15% of NeuN+ cells are tagged by Rbp4‐Cre::tdTomato (Hoerder‐Suabedissen et al., 2019) highlighting that the majority of L5 glutamatergic neurons are unaffected by the ablation of *Snap25*. Considering that the rest of the non‐Rbp4‐Cre + pyramidal neurons remain capable of regulated vesicle release, we speculate that these L5 pyramidal neurons may compensate for the cessation of activity of Rbp4‐Cre + L5 projection neurons. Moreover, we have not observed a significant loss in the density of the cell bodies of Rbp4‐Cre + neurons at P21, and a notable decline in Rbp4‐Cre + neurons was only noted at 8 months of age (Hoerder‐Suabedissen et al., 2019). This might explain why PV interneurons survive the chronic abolition of synaptic transmission and remain unaffected in cortical and subcortical regions as well.

Cortical layer 5 neurons have unique reciprocal connections with the thalamus and selectively innervate the higher‐order thalamic nuclei (Mo & Sherman, 2018; Hoerder‐Suabedissen et al., 2019; Hayashi et al., 2021; Casas‐Torremocha et al., 2022; Zolnik et al., 2024). The lateral posterior nucleus of the thalamus (LP) is one of the higher‐order thalamic nuclei innervated by L5 axon terminals and therefore, appeared to be a good candidate region for assessing the global effect of silencing L5 on the density of PV+ neurons. But neither in the developing nor in the adult brain did PV+ neurons in LP or the mediodorsal (MD) nucleus of the thalamus exhibit significant changes in their density. Regarding the thalamus, the ventral posteromedial nucleus of the thalamus (VPm) is found to be the single biggest source of input for the three canonical interneuron types (Hafner et al., 2019; Wall et al., 2016). However, the scarce number of PV neurons present in the rodent thalamus makes the investigation of the interaction between the thalamic projections of L5 and PV challenging.

The lack of effect of the chronic manipulation of L5 on PV density in the adult brain indicates that alterations in PV cell number are highly unlikely once the final number of GABAergic interneurons is determined during early development. However, their laminar positioning can still be influenced by chronically perturbing L5 pyramidal neurons. Since parvalbumin is activity‐dependent (Patz et al., 2004), we would argue that density changes are not driven by cell loss, but by a dynamic up‐ or downregulation of the parvalbumin protein that is responding to changes in the cortical network and changes in pyramidal neuron activity. This is supported by recent research showing that the parvalbumin‐expressing GABA interneurons are not eliminated in a mouse model of autism spectrum disorder and the downregulation of the parvalbumin protein expression is the cause of the apparent cell loss (Filice et al., 2016). The results of our recent study agree with this given that no major alterations were noted in the density of PV+ and VVA+ cells following the acute chemogenetic manipulation of Rbp4‐Cre + neurons (Vadisiute et al., 2024).

Previous studies have demonstrated that fate‐converting subcerebral projection neurons to callosal projection neurons altered the laminar distribution of GABAergic interneurons pointing to the role of glutamatergic pyramidal neuron identity in instructing the laminar arrangement of inhibitory neurons (Lodato et al., 2011; Wester et al., 2019; Wu et al., 2024 preprint). In our study, the chronic abolition of regulated vesicle release from L5 projection neurons changed the distribution of PV neurons in M1 and S1 at 3 months of age. We found a significant increase in the density of PV neurons in layer 4 of the primary somatosensory cortex. We also distinguished several subpopulations of interneurons given that there were PV cells that were not enwrapped by PNNs (PV+ VVA‐) and there were PNNs that did not colocalise with PV (PV‐ VVA+). It has been proposed that PV interneurons may orchestrate the organisation and the state of PNNs surrounding them as transcriptomic analysis revealed that many of the necessary PNN lecticans and linkers, as well as membrane and secretory proteases, are expressed by PV neurons (Devienne et al., 2021; Dityatev et al., 2007, 2010; Ferrer‐Ferrer & Dityatev, 2018; Kwok et al., 2012). The various subtypes of PV neurons might reflect the individual response of PV neurons to pyramidal neuron activity as they up or downregulate their PNNs depending on the network state. It is probable that the PV+ VVA‐ subtype is more plastic and so retains its ability to change to network disturbances while the PV+ VVA+ subtype has limited plasticity as it is consolidated by PNNs. Therefore, the PNNs may endow PV neurons with distinct properties depending on whether they are surrounded by proteoglycans or not. Previous studies have discovered that through its control on potassium channel localisation and synaptic AMPA receptor levels, brevican alters the intrinsic characteristics of PV+ interneurons and modulates PV function and excitability (Favuzzi et al., 2017).

The significant increase in the density of PV neurons in layer 4 of S1 at 3 months of age may indicate that the chronic cessation of L5 activity has a permanent effect on PV lamination that only manifests in adulthood. The concomitant increase in the density of PV+ VVA+ neurons in L4 may also suggest that PV interneurons try to counterbalance the absence of pyramidal neuron activity by upregulating the perineuronal net expression around them. Previous studies provide evidence that there is a direct correlation between PNN expression and thalamic innervation in primary sensory cortical regions (Lupori et al., 2023). It has also been shown that PNNs have the capacity to regulate thalamic afferents onto PV neurons and modulate visual input by thalamic recruitment of cortical PV interneurons (Faini et al., 2018). Our results show that chronically silencing L5 led to a significant decrease in the density of VVA+ and PV‐ VVA+ cells in L5 of M1 at 3 months of age. The alterations in the laminar arrangement of VVA+ neurons imply that the PNNs can be modulated by activity‐dependent mechanisms and Ca^2+^−dependent synaptic transmission may control PNN density in adult networks. The observed reductions in VVA+ in L5 of M1 may imply that adult cortical circuits are more receptive to high circuit plasticity when PNNs are decreased. The different patterns in the alterations of the laminar density of PV+ and VVA+ cells suggest that the influence of pyramidal cell activity on GABAergic interneurons and PNNs is regional and subtype‐specific. Our results further support the regional and subtype‐specific differences in interneuron density reported by previous studies (De Marco García et al., 2011; Duan et al., 2020; Pouchelon et al., 2021; Ueno et al., 2018).

Remarkably, we observed that the correlation between PV and VVA was altered in the adult *Snap25* cKO mice in M1. Layer 2/3 and layer 5 in M1 had a reverse correlation between PV and VVA in the *Snap25*‐ablated mice. There was a high degree of correlation between PV and VVA density in layer 6a in S1. However, cortical layer 5 and layer 6b of S1 revealed a negative correlation between PV and VVA density. These findings support prior studies that demonstrate the association between PV and PNN is extremely region‐dependent, fluctuates throughout layers, and differs significantly between cortical areas (Lupori et al., 2023).

One of the major limitations of the present study is that the Rbp4‐Cre is a pan L5 line and therefore, it is not truly restrictive to a specific subset of L5 pyramidal neurons. Since the Rbp4‐Cre line is unable to differentiate between IT vs. ET‐type L5 projection neurons, other transgenic models with more precise genetic targeting of L5 pyramidal neuronal subgroups will be needed. Another limiting factor is the chronic manipulation of pyramidal neuron activity. The *Rbp4‐Cre*;*Snap25* cKO mice only permit the permanent and irreversible manipulation of pyramidal neuron activity. Engineering of new CreERT mouse lines will allow us to manipulate pyramidal neurons in an acute fashion and perturb pyramidal neuron function with temporal precision.

In this study, we performed a chronic, irreversible manipulation of subsets of L5 projection neurons to unveil the role of deep‐layer pyramidal neurons in instructing the laminar and spatial distribution of PV neurons. We demonstrated the absence of chronic vesicle release from L5 projection neurons allows for the normal development of PV+ neurons, but it leads to a redistribution of PV+ and VVA+ neurons in a region‐and layer‐specific manner. It appears that distinct subtypes of PV neurons rely on different survival signals and region‐specific mechanisms govern PV function and number. Further studies examining PV and PNN interactions will uniquely inform us about the plasticity of cortical circuits, the differential survival capacity of inhibitory interneurons and the pathophysiological changes underpinning neuropsychiatric disorders with interneuronopathies and synaptopathies.

## AUTHOR CONTRIBUTIONS

Z.M. and A.H.S. conceptualised and designed the study. F.SZ. performed immunohistochemistry experiments, morphometrics analyses, density and laminar distribution analyses, correlation analyses and imaging, and wrote the manuscript. V.S. performed Vglut1 IHC experiments. Z.M. and A.H.S. supervised the project and secured funding. A.M.S., V.S. and A.H.S. performed pilot experiments.

## CONFLICT OF INTEREST STATEMENT

The authors declare no conflicts of interest.

## Supporting information


Table S1.

## Data Availability

The data that support the findings of this study are available from the corresponding authors upon request.
