# Supplementary material for: Chronic silencing of subsets of cortical layer 5 pyramidal neurons has a long‐term influence on the laminar distribution of parvalbumin interneurons and the perineuronal nets
Source: J Anat. 2024 Dec 3;246(4):479–504. doi: 10.1111/joa.14181 (PMC11911141; doi:10.1111/joa.14181)
Supplement: Supplementary file 1 — Table S1. [file JOA-246-479-s001.docx]

**Supplementary materials**

| **Transgenic**  **strain** | **Mouse**  **Genotype** | **Experimental**  **Cohort** | **Timepoint** | **Nr of brains** | **Nr of sections** | **IHC** |
| --- | --- | --- | --- | --- | --- | --- |
| *Rbp4-Cre;Ai14;Snap25^fl/fl^* | Cre-;Ai14;Snap25^fl/fl^ or  Cre-;Ai14;Snap25^fl/+^ (ctrl) | Developmental | P14 | 3 | 4 | *Parvalbumin*  *Vglut1* |
| *Rbp4-Cre;Ai14;Snap25^fl/fl^* | Cre+;Ai14;Snap25^fl/fl^ (cKO) | Developmental | P14 | 3 | 4 | *Parvalbumin*  *Vglut1* |
| *Rbp4-Cre;Ai14;Snap25^fl/fl^* | Cre-;Ai14;Snap25^fl/fl^ or  Cre-;Ai14;Snap25^fl/+^ (ctrl) | Developmental | P21 | 4 | 4 | *Parvalbumin*  *Vglut1* |
| *Rbp4-Cre;Ai14;Snap25^fl/fl^* | Cre+;Ai14;Snap25^fl/fl^ (cKO) | Developmental | P21 | 4 | 4 | *Parvalbumin*  *Vglut1* |
| *Rbp4-Cre;Ai14;Snap25^fl/fl^* | Cre-;Ai14;Snap25^fl/fl^ or  Cre-;Ai14;Snap25^fl/+^ (ctrl) | Adult | 12 weeks | 5 | 3 | *Parvalbumin*  *Vicia villosa* |
| *Rbp4-Cre;Ai14;Snap25^fl/fl^* | Cre+;Ai14;Snap25^fl/fl^ (cKO) | Adult | 12 weeks | 5 | 3 | *Parvalbumin*  *Vicia villosa* |
| *Both male and female mice were used throughout the experiments.* | | | | | | |

**Supplementary Table 1**. Number of brains and number of sections used per experimental cohorts and genotype to determine the density, laminar distribution, developmental trajectory, and Pearson correlation between PV and VVA neurons. The table also indicates the type of immunohistochemical staining performed at each time point.

| **Transgenic**  **strain** | **Mouse**  **Genotype** | **Experimental**  **Cohort** | **Timepoint** | **Nr of cells** | **Nr of sections** | **Measured**  **features** |
| --- | --- | --- | --- | --- | --- | --- |
| *Rbp4-Cre;Ai14;Snap25^fl/fl^* | Cre-;Ai14;Snap25^fl/fl^ or  Cre-;Ai14;Snap25^fl/+^ (ctrl) | Developmental | P14 | 175 | 4 | *Soma area*  *Perimeter*  *Circularity*  *Feret*  *MinFeret*  *Feret angle*  *Roundness*  *Solidity* |
| *Rbp4-Cre;Ai14;Snap25^fl/fl^* | Cre+;Ai14;Snap25^fl/fl^ (cKO) | Developmental | P14 | 131 | 4 |  |
| *Rbp4-Cre;Ai14;Snap25^fl/fl^* | Cre-;Ai14;Snap25^fl/fl^ or  Cre-;Ai14;Snap25^fl/+^ (ctrl) | Developmental | P21 | 180 | 4 |  |
| *Rbp4-Cre;Ai14;Snap25^fl/fl^* | Cre+;Ai14;Snap25^fl/fl^ (cKO) | Developmental | P21 | 236 | 4 |  |
| *Rbp4-Cre;Ai14;Snap25^fl/fl^* | Cre-;Ai14;Snap25^fl/fl^ or  Cre-;Ai14;Snap25^fl/+^ (ctrl) | Adult | 12 weeks | 168 | 3 |  |
| *Rbp4-Cre;Ai14;Snap25^fl/fl^* | Cre+;Ai14;Snap25^fl/fl^ (cKO) | Adult | 12 weeks | 171 | 3 |  |
| *Both male and female mice were used throughout the experiments.* | | | | | | |

**Supplementary Table 2**. Number of cells and number of sections used to perform morphometric analyses of various soma features of PV-positive neurons in the caudoputamen at each time point. The table shows the soma parameters selected for morphometric measurements.

| **Transgenic**  **strain** | **Mouse**  **Genotype** | **Experimental**  **Cohort** | **Time**  **point** | **Cortical ROIs**  **(Local effect)** | **Subcortical ROIs**  **(Global effect)** | **Morphometrics** | **Pearson correlation** |
| --- | --- | --- | --- | --- | --- | --- | --- |
| *Rbp4-Cre;Ai14;Snap25^fl/fl^* | Cre-;Ai14;Snap25^fl/fl^ or  Cre-;Ai14;Snap25^fl/+^ (ctrl) | Developmental | P14 | Primary motor cortex (M1)  Primary somatosensory cortex (S1) | Caudoputamen (CPu)  Globus pallidus, external segment (GPe)  Lateral posterior nucleus of the thalamus (LP)  Mediodorsal nucleus of the thalamus (MD)  Superior colliculus (SC) | Caudoputamen (CPu) | Primary motor cortex (M1)  Primary somatosensory cortex (S1)  Caudoputamen (CPu) |
| *Rbp4-Cre;Ai14;Snap25^fl/fl^* | Cre+;Ai14;Snap25^fl/fl^ (cKO) | Developmental | P14 |  |  |  |  |
| *Rbp4-Cre;Ai14;Snap25^fl/fl^* | Cre-;Ai14;Snap25^fl/fl^ or  Cre-;Ai14;Snap25^fl/+^ (ctrl) | Developmental | P21 |  |  |  |  |
| *Rbp4-Cre;Ai14;Snap25^fl/fl^* | Cre+;Ai14;Snap25^fl/fl^ (cKO) | Developmental | P21 |  |  |  |  |
| *Rbp4-Cre;Ai14;Snap25^fl/fl^* | Cre-;Ai14;Snap25^fl/fl^ or  Cre-;Ai14;Snap25^fl/+^ (ctrl) | Adult | 12 weeks |  |  |  |  |
| *Rbp4-Cre;Ai14;Snap25^fl/fl^* | Cre+;Ai14;Snap25^fl/fl^ (cKO) | Adult | 12 weeks |  |  |  |  |

**Supplementary Table 3**. Cortical and subcortical regions of interest selected to examine the *local* (location of the cell bodies of Rbp4-Cre+ L5 neurons) and the *global* (projection sites of Rbp4-Cre+ L5 neurons) effects of chronically abolishing regulated synaptic vesicle release from cortical L5 projection neurons. The table also indicates the regions of interest for morphometric analyses of PV neurons and for computing the Pearson correlation between PV and VVA.

| **Morphometrics** | | | |
| --- | --- | --- | --- |
| **Parvalbumin *- Genotype* effect** | | | |
| Development | | | Adult |
| **Parameters measured** | **P14 ctrl - cKO** | **P21 ctrl - cKO** | **12 weeks ctrl - cKO** |
| Soma Area | ns | * | ns |
| Soma Perimeter | ns | ns | ns |
| Soma Circularity | ns | ns | ns |
| Soma Roundness | ns | ** | ns |
| Soma Solidity | ns | ns | ns |

**Supplementary Table 4**. Summary table showing the effect of genotype on the soma morphology of parvalbumin neurons in the caudoputamen. Morphometric analyses were performed to determine the short- and long-term impacts of chronically silencing L5 projection neurons on the soma morphology of PV interneurons.

| **Morphometrics** | | | | |
| --- | --- | --- | --- | --- |
| **Parvalbumin *- Trajectory*** | | | | |
|  | Development | | Adult | |
| **Parameters**  **measured** | **P14 - P21 ctrl** | **P14 -P21 cKO** | **P21 - Adult ctrl** | **P21 - Adult cKO** |
| Soma Area | ns | *** | **** | ** |
| Soma Perimeter | ns | ns | ns | ns |
| Soma Circularity | ns | ns | **** | ** |
| Soma Roundness | ns | *** | ** | ns |
| Soma Solidity | ns | ns | **** | **** |

**Supplementary Table 5**. Summary table showing how the soma morphology of parvalbumin neurons in the caudoputamen changes over time in the control and the layer 5-silenced cKO mice. Morphometric analyses were conducted to determine if the soma morphology of PV neurons deviates from that of the control mice.

**Supplementary Table 6.** Summary of the results of the density, laminar distribution, and trajectory of PV neurons and their different subpopulations (PV+ VVA+, PV+ VVA-, PV- VVA+) in the cortical and subcortical regions of interest at different postnatal stages and in adulthood.

| **Figure** | **Analysis** | **Effect** | **Values** | **Statistical test** | **P value** |
| --- | --- | --- | --- | --- | --- |
| **Figure 3f** | PV interneuron  *cell density* (mean ± SEM) | ***Genotype***  (Local) | P14 M1 ctrl: 93.561 ± 3.589 | two-way ANOVA with Šídák's multiple comparisons test | p=0.3679 (ns) |
|  |  |  | P14 M1 cKO: 135.431 ± 40.920 |  |  |
|  |  |  | P21 M1 ctrl: 105.176 ± 5.746 | two-way ANOVA with Šídák's multiple comparisons test | p=0.6323 (ns) |
|  |  |  | P21 M1 cKO: 129.017 ± 17.756 |  |  |
| **Figure 3g** | PV interneuron  *cell density* (mean ± SEM) | ***Genotype***  (Local) | P14 S1 ctrl: 130.780 ± 13.257 | two-way ANOVA with Šídák's multiple comparisons test | p=0.5806 (ns) |
|  |  |  | P14 S1 cKO: 168.428 ± 47.865 |  |  |
|  |  |  | P21 S1 ctrl: 139.705 ± 16.128 | two-way ANOVA with Šídák's multiple comparisons test | p=0.8327 (ns) |
|  |  |  | P21 S1 cKO: 158.262 ± 19.200 |  |  |
| **Figure 3h** | PV interneuron  cell density  (mean ± SEM) | ***Trajectory***  (Local) | P14 M1 ctrl: 93.561 ± 3.589 | two-way ANOVA with Šídák's multiple comparisons test | p=0.7467 (ns) |
|  |  |  | P21 M1 ctrl: 105.176 ± 5.746 |  |  |
|  |  |  | P14 S1 ctrl: 130.780 ± 13.257 | two-way ANOVA with Šídák's multiple comparisons test | p= 0.8398 (ns) |
|  |  |  | P21 S1 ctrl: 139.705 ± 16.128 |  |  |
| **Figure 3i** | PV interneuron  cell density  (mean ± SEM) | ***Trajectory***  (Local) | P14 M1 cKO: 135.431 ± 40.920 | two-way ANOVA with Šídák's multiple comparisons test | p= 0.9868 (ns) |
|  |  |  | P21 M1 cKO: 129.017 ± 17.756 |  |  |
|  |  |  | P14 S1 cKO: 168.428 ± 47.865 | two-way ANOVA with Šídák's multiple comparisons test | p= 0.9671 (ns) |
|  |  |  | P21 S1 cKO: 158.262 ± 19.200 |  |  |
| **Figure**  **4c** | PV interneuron  cell density  (mean ± SEM) | ***Genotype***  (Global) | P14 LP ctrl: 50.4 ± 5.9,  P14 LP cKO: 64.4 ± 28 | two-way ANOVA with Šídák's multiple comparisons test | p=0.999  (ns) |
|  |  |  | P14 MD ctrl: 59.6 ± 4.6,  P14 MD cKO: 48.0 ± 5.2 | two-way ANOVA with Šídák's multiple comparisons test | p= >0.9999  (ns) |
|  |  |  | P14 CPu ctrl: 84.4 ± 6.6  P14 CPu cKO: 69.7 ± 3.4 | two-way ANOVA with Šídák's multiple comparisons test | p=0.9998  (ns) |
|  |  |  | P14 GPe ctrl: 381.1 ± 39.4 P14 GPe cKO: 420.3 ± 71.8 | two-way ANOVA with Šídák's multiple comparisons test | p= 0.9813  (ns) |
|  |  |  | P14 SC ctrl: 246.6 ± 40.3 P14 SC cKO: 355.3 ± 106.8 | two-way ANOVA with Šídák's multiple comparisons test | p= 0.4308  (ns) |
| **Figure**  **4d** | PV interneuron  cell density  (mean ± SEM) | ***Genotype***  (Global) | P21 LP ctrl: 50.2 ± 14.0  P21 LP cKO: 63.6 ± 10.0 | two-way ANOVA with Šídák's multiple comparisons test | p= >0.9999  (ns) |
| **Figure**  **4d** | PV interneuron  cell density  (mean ± SEM) | ***Genotype***  (Global) | P21 MD ctrl: 62.8 ± 1.6 P21 P21 MD cKO: 64.3 ± 10.2 | two-way ANOVA with Šídák's multiple comparisons test | p= >0.9999  (ns) |
|  |  |  | P21 CPu ctrl: 86.3 ± 5.1  P21 CPu cKO: 85.3 ± 4.9 | two-way ANOVA with Šídák's multiple comparisons test | p= >0.9999  (ns) |
|  |  |  | P21 GPe ctrl: 397.0 ± 50.8  P21 GPe cKO: 485.0 ± 117.4 | two-way ANOVA with Šídák's multiple comparisons test | p= 0.7598  (ns) |
|  |  |  | P21 SC ctrl: 438.7 ± 94.8  P21 SC cKO: 415.7 ± 45.9 | two-way ANOVA with Šídák's multiple comparisons test | p= 0.9992  (ns) |
| **Figure**  **4e** | PV interneuron  cell density  (mean ± SEM) | ***Trajectory***  (Global) | P14 LP ctrl: 50.4 ± 5.9 | two-way ANOVA with Šídák's multiple comparisons test | p= >0.9999  (ns) |
|  |  |  | P21 LP ctrl : 50.2 ± 14.0 |  |  |
|  |  |  | P14 MD ctrl: 59.6 ± 4.6 | two-way ANOVA with Šídák's multiple comparisons test | p= >0.9999  (ns) |
|  |  |  | P21 MD ctrl: 62.8 ± 1.6 |  |  |
|  |  |  | P14 CPu ctrl: 84.4 ± 6.6 | two-way ANOVA with Šídák's multiple comparisons test | p= >0.9999  (ns) |
|  |  |  | P21 CPu ctrl: 87.0 ± 5.4 |  |  |
|  |  |  | P14 GPe ctrl: 381.1 ± 39.4 | two-way ANOVA with Šídák's multiple comparisons test | p= 0.9997  (ns) |
|  |  |  | P21 GPe ctrl: 397.0 ± 50.8 |  |  |
|  |  |  | P14 SC ctrl: 246.6 ± 40.3 | two-way ANOVA with Šídák's multiple comparisons test | p= 0.0217  (*) |
|  |  |  | P21 SC ctrl: 438.7 ± 94.8 |  |  |
| **Figure**  **4f** | PV interneuron  cell density  (mean ± SEM) | ***Trajectory***  (Global) | P14 LP cKO: 64.4 ± 28.5 | two-way ANOVA with Šídák's multiple comparisons test | p= >0.9999  (ns) |
|  |  |  | P21 LP cKO: 63.6 ± 10.0 |  |  |
|  |  |  | P14 MD cKO: 48.0 ± 5.2 | two-way ANOVA with Šídák's multiple comparisons test | p= >0.9999  (ns) |
|  |  |  | P21 MD cKO: 64.3 ± 10.2 |  |  |
|  |  |  | P14 CPu cKO: 69.7 ± 3.4 | two-way ANOVA with Šídák's multiple comparisons test | p= >0.9999  (ns) |
|  |  |  | P21 CPu cKO: 85.3 ± 4.9 |  |  |
|  |  |  | P14 GPe cKO: 420.3 ± 71.8 | two-way ANOVA with Šídák's multiple comparisons test | p= 0.9474  (ns) |
|  |  |  | P21 GPe cKO: 485.0 ± 117.3 |  |  |
|  |  |  | P14 SC cKO: 355.3 ± 106.8 | two-way ANOVA with Šídák's multiple comparisons test | p= 0.9605  (ns) |
|  |  |  | P21 SC cKO: 415.7 ± 45.9 |  |  |
| **Figure**  **5d** | PV interneuron  soma measurements  (mean ± SEM) | ***Genotype***  ***Morphology***  (Global) | *Soma Area*  P14 CPu ctrl: 172.6 ± 5.5  P14 CPu cKO: 174.7 ± 6.4 | unpaired student t-test with Welch's correction | p= 0.8085  (ns) |
|  |  |  | *Soma Perimeter*  P14 CPu ctrl: 58.6 ± 1.6  P14 CPu cKO: 60.5 ± 2.3 | unpaired student t-test with Welch's correction | p= 0.5071  (ns) |
|  |  |  | *Soma Circularity*  P14 CPu ctrl: 0.663 ± 0.0  P14 CPu cKO: 0.656 ± 0.0 | unpaired student t-test with Welch's correction | p= 0.7071  (ns) |
| **Figure**  **5d** | PV interneuron  soma measurements  (mean ± SEM) | ***Genotype***  ***Morphology***  (Global) | *Soma Roundness*  P14 CPu ctrl: 0.695 ± 0.0  P14 CPu cKO: 0.712 ± 0.0 | unpaired student t-test with Welch's correction | p= 0.2717  (ns) |
|  |  |  | *Soma Solidity*  P14 CPu ctrl: 0.865 ± 0.0  P14 CPu cKO: 0.851 ± 0.0 | unpaired student t-test with Welch's correction | p= 0.2479  (ns) |
| **Figure**  **5f** | PV interneuron  soma measurements  (mean ± SEM) | ***Genotype***  ***Morphology***  (Global) | *Soma Area*  P21 CPu ctrl: 161.7 ± 4.7  P21 CPu cKO: 147.1 ± 4.0 | unpaired student t-test with Welch's correction | p= 0.0192  (*) |
|  |  |  | *Soma Perimeter*  P21 CPu ctrl: 57.9 ± 1.6  P21 CPu cKO: 55.3 ± 1.4 | unpaired student t-test with Welch's correction | p= 0.2311  (ns) |
|  |  |  | *Soma Circularity*  P21 CPu ctrl: 0.648 ± 0.0  P21 CPu cKO: 0.647 ± 0.0 | unpaired student t-test with Welch's correction | p= 0.9626  (ns) |
|  |  |  | *Soma Roundness*  P21 CPu ctrl: 0.701 ± 0.0  P21 CPu cKO: 0.661 ± 0.0 | unpaired student t-test with Welch's correction | p= 0.0035  (**) |
|  |  |  | *Soma Solidity*  P21 CPu ctrl: 0.849 ± 0.0  P21 CPu cKO: 0.852 ± 0.0 | unpaired student t-test with Welch's correction | p= 0.7592  (ns) |
| **Figure**  **6b** | PV interneuron  soma measurements  (mean ± SEM) | ***Trajectory***  ***Morphology***  (Global) | *Soma Area*  P14 CPu ctrl: 172.6 ± 5.5  P21 CPu ctrl: 161.7 ± 4.7 | unpaired student t-test with Welch's correction | p=0.1335  (ns) |
|  |  |  | *Soma Perimeter*  P14 CPu ctrl: 58.6 ± 1.6  P21 CPu ctrl: 57.9 ± 1.6 | unpaired student t-test with Welch's correction | p=0.7445  (ns) |
|  |  |  | *Soma Circularity*  P14 CPu ctrl: 0.663 ± 0.0  P21 CPu ctrl: 0.648 ± 0.0 | unpaired student t-test with Welch's correction | p=0.3908  (ns) |
|  |  |  | *Soma Roundness*  P14 CPu ctrl: 0.695 ± 0.0  P21 CPu ctrl: 0.701 ± 0.0 | unpaired student t-test with Welch's correction | p=0.7074  (ns) |
|  |  |  | *Soma Solidity*  P14 CPu ctrl: 0.865 ± 0.0  P21 CPu ctrl: 0.849 ± 0.0 | unpaired student t-test with Welch's correction | p=0.1297  (ns) |
| **Figure**  **6d** | PV interneuron  soma measurements  (mean ± SEM) | ***Trajectory***  ***Morphology***  (Global) | *Soma Area*  P14 CPu cKO: 174.7 ± 6.4  P21 CPu cKO: 147.1 ± 4.0 | unpaired student t-test with Welch's correction | p= 0.0003  (***) |
|  |  |  | *Soma Perimeter*  P14 CPu cKO: 60.5 ± 2.3  P21 CPu cKO: 55.3 ± 1.4 | unpaired student t-test with Welch's correction | p= 0.0558  (ns) |
|  |  |  | *Soma Circularity*  P14 CPu cKO: 0.656 ± 0.0  P21 CPu cKO: 0.647 ± 0.0 | unpaired student t-test with Welch's correction | p=0.6464  (ns) |
|  |  |  | *Soma Roundness*  P14 CPu cKO: 0.712 ± 0.0  P21 CPu cKO: 0.661 ± 0.0 | unpaired student t-test with Welch's correction | p= 0.0003  (***) |
|  |  |  | *Soma Solidity*  P14 CPu cKO: 0.851 ± 0.0  P21 CPu cKO: 0.852 ± 0.0 | unpaired student t-test with Welch's correction | p= 0.9262  (ns) |
| **Figure**  **7b** | PV interneuron  soma measurements  (mean ± SEM) | ***Genotype***  ***Morphology***  (Global) | *Soma Area*  12 weeks CPu ctrl: 121 ± 3.8  12 weeks CPu cKO: 129 ± 4.7 | unpaired student t-test with Welch's correction | p= 0.2301  (ns) |
|  |  |  | *Soma Perimeter*  12 weeks CPu ctrl: 56.8 ± 1.9  12 weeks CPu cKO: 56.6 ± 2.1 | unpaired student t-test with Welch's correction | p= 0.9398  (ns) |
|  |  |  | *Soma Circularity*  12 weeks CPu ctrl: 0.555 ± 0.0  12 weeks CPu cKO: 0.585 ± 0.0 | unpaired student t-test with Welch's correction | p= 0.1721  (ns) |
|  |  |  | *Soma Roundness*  12 weeks CPu ctrl: 0.656 ± 0.0  12 weeks CPu cKO: 0.669 ± 0.0 | unpaired student t-test with Welch's correction | p= 0.4804  (ns) |
|  |  |  | *Soma Solidity*  12 weeks CPu ctrl: 0.782 ± 0.0  12 weeks CPu cKO: 0.797 ± 0.0 | unpaired student t-test with Welch's correction | p= 0.3412  (ns) |
| **Figure**  **7d** | PV interneuron  soma measurements  (mean ± SEM) | ***Trajectory***  ***Morphology***  (Global) | *Soma Area*  P21 CPu ctrl: 161.7 ± 4.7  12 weeks CPu ctrl: 121 ± 3.8 | unpaired student t-test with Welch's correction | p= <0.0001  (****) |
|  |  |  | *Soma Perimeter*  P21 CPu ctrl: 57.9 ± 1.6  12 weeks CPu ctrl: 56.8 ± 1.9 | unpaired student t-test with Welch's correction | p= 0.6702  (ns) |
|  |  |  | *Soma Circularity*  P21 CPu ctrl: 0.648 ± 0.0  12 weeks CPu ctrl: 0.555 ± 0.0 | unpaired student t-test with Welch's correction | p= <0.0001  (****) |
| **Figure**  **7d** | PV interneuron  soma measurements  (mean ± SEM) | ***Trajectory***  ***Morphology***  (Global) | *Soma Roundness*  P21 CPu ctrl: 0.701 ± 0.0  12 weeks CPu ctrl: 0.656 ± 0.0 | unpaired student t-test with Welch's correction | p= 0.0050  (**) |
|  |  |  | *Soma Solidity*  P21 CPu ctrl: 0.849 ± 0.0  12 weeks CPu ctrl: 0.782 ± 0.0 | unpaired student t-test with Welch's correction | p= <0.0001  (****) |
| **Figure**  **7e** | PV interneuron  soma measurements  (mean ± SEM) | ***Trajectory***  ***Morphology***  (Global) | *Soma Area*  P21 CPu cKO: 147.1 ± 4.0  12 weeks CPu cKO: 129 ± 4.7 | unpaired student t-test with Welch's correction | p= 0.0028  (**) |
|  |  |  | *Soma Perimeter*  P21 CPu cKO: 55.3 ± 1.4  12 weeks CPu cKO: 56.6 ± 2.1 | unpaired student t-test with Welch's correction | p= 0.6048  (ns) |
|  |  |  | *Soma Circularity*  P21 CPu cKO: 0.647 ± 0.0  12 weeks CPu cKO: 0.585 ± 0.0 | unpaired student t-test with Welch's correction | p= 0.0020  (**) |
|  |  |  | *Soma Roundness*  P21 CPu cKO: 0.661 ± 0.0  12 weeks CPu cKO: 0.669 ± 0.0 | unpaired student t-test with Welch's correction | p= 0.6391  (ns) |
|  |  |  | *Soma Solidity*  P21 CPu cKO: 0.852 ± 0.0  12 weeks CPu cKO: 0.797 ± 0.0 | unpaired student t-test with Welch's correction | p= <0.0001  (****) |
| **Figure**  **8c** | PV interneuron  distribution  (mean ± SEM) | ***Genotype***  ***Laminar Distribution***  (Local) | **P14 M1**  L1: 0 ± 0 (ctrl),  L1: 0 ± 0 (cKO) | two-way ANOVA with Šídák's multiple comparisons test | p= >0.9999  (ns) |
|  |  |  | L2/3: 106.8 ± 6.5 (ctrl), L2/3: 164.7 ± 52.9 (cKO) | two-way ANOVA with Šídák's multiple comparisons test | p= 0.5340  (ns) |
|  |  |  | L5: 129.3 ± 5.6 (ctrl),  L5: 174.7 ± 43.1 (cKO) | two-way ANOVA with Šídák's multiple comparisons test | p= 0.7345  (ns) |
|  |  |  | L6: 59.0 ± 8.2 (ctrl),  L6: 87.1 ± 42.8 (cKO) | two-way ANOVA with Šídák's multiple comparisons test | p= 0.9370  (ns) |
| **Figure**  **8d** | PV interneuron  distribution  (mean ± SEM) | ***Genotype***  ***Laminar Distribution***  (Local) | **P21 M1**  L1: 0 ± 0 (ctrl),  L1: 0 ± 0 (cKO) | two-way ANOVA with Šídák's multiple comparisons test | p= >0.9999  (ns) |
|  |  |  | L2/3: 126.8 ± 9.7 (ctrl), L2/3: 150.3 ± 22.4 (cKO) | two-way ANOVA with Šídák's multiple comparisons test | p= 0.6876  (ns) |
| **Figure**  **8d** | PV interneuron  distribution  (mean ± SEM) | ***Genotype***  ***Laminar Distribution***  (Local) | L5: 145.2 ± 9.8 (ctrl),  L5: 170.0 ± 25.1 (cKO) | two-way ANOVA with Šídák's multiple comparisons test | p= 0.6454  (ns) |
|  |  |  | L6: 38.8 ± 10.6 (ctrl),  L6: 68.7 ± 13.0 (cKO) | two-way ANOVA with Šídák's multiple comparisons test | p= 0.4757  (ns) |
| **Figure**  **8e** | PV interneuron  distribution  (mean ± SEM) | ***Trajectory***  ***Laminar Distribution***  (Local) | **P14-P21 M1**  L1: 0 ± 0 (P14, ctrl),  L1: 0 ± 0 (P21, ctrl) | two-way ANOVA with Šídák's multiple comparisons test | p=>0.9999  (ns) |
|  |  |  | L2/3: 106.8 ± 6.5 (P14, ctrl), L2/3: 126.8 ± 9.7 (P21, ctrl) | two-way ANOVA with Šídák's multiple comparisons test | p=0.3337  (ns) |
|  |  |  | L5: 129.3 ± 5.6 (P14, ctrl), L5: 145.2 ± 9.9 (P21, ctrl) | two-way ANOVA with Šídák's multiple comparisons test | p=0.5500  (ns) |
|  |  |  | L6: 59.0 ± 8.2 (P14, ctrl),  L6: 38.8 ± 10.6 (P21, ctrl) | two-way ANOVA with Šídák's multiple comparisons test | p=0.3260  (ns) |
| **Figure**  **8f** | PV interneuron  distribution  (mean ± SEM) | ***Trajectory***  ***Laminar Distribution***  (Local) | **P14-P21 M1**  L1: 0 ± 0 (P14, cKO),  L1: 0 ± 0 (P21, cKO) | two-way ANOVA with Šídák's multiple comparisons test | p=>0.9999  (ns) |
|  |  |  | L2/3: 164.7 ± 52.9 (P14, cKO),  L2/3: 150.3 ± 22.4 (P21, cKO) | two-way ANOVA with Šídák's multiple comparisons test | p=0.9939  (ns) |
|  |  |  | L5: 174.7 ± 43.1 (P14, cKO), L5: 170.0 ± 25.1 (P21, cKO) | two-way ANOVA with Šídák's multiple comparisons test | p=>0.9999  (ns) |
|  |  |  | L6: 87.1 ± 42.8 (P14, cKO), L6: 68.7 ± 13.0 (P21, cKO) | two-way ANOVA with Šídák's multiple comparisons test | p=0.9849  (ns) |
| **Figure**  **8i** | PV interneuron  distribution  (mean ± SEM) | ***Genotype***  ***Laminar Distribution***  (Local) | **P14 S1**  L1: 0 ± 0 (ctrl),  L1: 0 ± 0 (cKO) | two-way ANOVA with Šídák's multiple comparisons test | p=>0.9999  (ns) |
|  |  |  | L2/3: 96.1 ± 19.0 (ctrl),  L2/3: 93.3 ± 29.2 (cKO) | two-way ANOVA with Šídák's multiple comparisons test | p=>0.9999  (ns) |
|  |  |  | L4: 336.1 ± 49.5 (ctrl),  L4: 390.1 ± 130.4 (cKO) | two-way ANOVA with Šídák's multiple comparisons test | p= 0.9541  (ns) |
| **Figure**  **8i** | PV interneuron  distribution  (mean ± SEM) | ***Genotype***  ***Laminar Distribution***  (Local) | L5: 186.3 ± 18.2 (ctrl),  L5: 243.2 ± 63.0 (cKO) | two-way ANOVA with Šídák's multiple comparisons test | p= 0.9434  (ns) |
|  |  |  | L6: 63.2 ± 5.6 (ctrl),  L6: 106.4 ± 26.9 (cKO) | two-way ANOVA with Šídák's multiple comparisons test | p= 0.9824  (ns) |
| **Figure**  **8j** | PV interneuron  distribution  (mean ± SEM) | ***Genotype***  ***Laminar Distribution***  (Local) | **P21 S1**  L1: 0 ± 0 (ctrl),  L1: 0 ± 0 (cKO) | two-way ANOVA with Šídák's multiple comparisons test | p=>0.9999  (ns) |
|  |  |  | L2/3: 122.3 ± 10.7 (ctrl), L2/3: 124.8 ± 13.7 (cKO) | two-way ANOVA with Šídák's multiple comparisons test | p= >0.9999  (ns) |
|  |  |  | L4: 352.6 ± 53.3 (ctrl),  L4: 307.1 ± 26.9 (cKO) | two-way ANOVA with Šídák's multiple comparisons test | p= 0.6483  (ns) |
|  |  |  | L5: 190.7 ± 21.1 (ctrl),  L5: 214.5 ± 23.3 (cKO) | two-way ANOVA with Šídák's multiple comparisons test | p= 0.9640  (ns) |
|  |  |  | L6: 68.8 ± 19.8 (ctrl),  L6: 99.4 ± 21.4 (cKO) | two-way ANOVA with Šídák's multiple comparisons test | p= 0.9024  (ns) |
| **Figure**  **8k** | PV interneuron  distribution  (mean ± SEM) | ***Trajectory***  ***Laminar Distribution***  (Local) | **P14-P21 S1**  L1: 0 ± 0 (P14, ctrl),  L1: 0 ± 0 (P21, ctrl) | two-way ANOVA with Šídák's multiple comparisons test | p=>0.9999  (ns) |
|  |  |  | L2/3: 96.1 ± 19.0 (P14, ctrl), L2/3: 122.3 ± 10.7 (P21, ctrl) | two-way ANOVA with Šídák's multiple comparisons test | p=0.9706  (ns) |
|  |  |  | L4: 336.1 ± 49.5 (P14, ctrl), L4: 352.6 ± 53.3 (P21, ctrl) | two-way ANOVA with Šídák's multiple comparisons test | p=0.9964  (ns) |
|  |  |  | L5: 186.3 ± 18.2 (P14, ctrl), L5: 190.7 ± 21.1 (P21, ctrl) | two-way ANOVA with Šídák's multiple comparisons test | p=>0.9999  (ns) |
|  |  |  | L6: 63.2 ± 5.6 (P14, ctrl),  L6: 68.8 ± 19.8 (P21, ctrl) | two-way ANOVA with Šídák's multiple comparisons test | p=>0.9999  (ns) |
| **Figure**  **8l** | PV interneuron  distribution  (mean ± SEM) | ***Trajectory***  ***Laminar Distribution***  (Local) | **P14-P21 S1**  L1: 0 ± 0 (P14, cKO),  L1: 0 ± 0 (P21, cKO) | two-way ANOVA with Šídák's multiple comparisons test | p=>0.9999  (ns) |
| **Figure**  **8l** | PV interneuron  distribution  (mean ± SEM) | ***Trajectory***  ***Laminar Distribution***  (Local) | L2/3: 93.3 ± 29.2 (P14, cKO),  L2/3: 124.8 ± 13.7 (P21, cKO) | two-way ANOVA with Šídák's multiple comparisons test | p= 0.9909  (ns) |
|  |  |  | L4: 390.1 ± 130.4 (P14, cKO),  L4: 307.1 ± 26.9 (P21, cKO) | two-way ANOVA with Šídák's multiple comparisons test | p=0.6379  (ns) |
|  |  |  | L5: 243.2 ± 62.9 (P14, cKO), L5: 214.5 ± 23.3 (P21, cKO) | two-way ANOVA with Šídák's multiple comparisons test | p= 0.9941  (ns) |
|  |  |  | L6: 106.4 ± 26.9 (P14, cKO), L6: 99.4 ± 21.4 (P21, cKO) | two-way ANOVA with Šídák's multiple comparisons test | p= >0.9999  (ns) |
| **Figure**  **9e** | PV interneuron  density  (mean ± SEM) | ***Genotype***  (Local) | **Adult (12-week-old)**  M1 ctrl: 109.3 ± 15.0 | two-way ANOVA with Šídák's multiple comparisons test | p= 0.9700  (ns) |
|  |  |  | M1 cKO: 105.0 ± 6.4 |  |  |
|  |  |  | S1 ctrl: 132.5 ± 14.6 | two-way ANOVA with Šídák's multiple comparisons test | p= 0.2887  (ns) |
|  |  |  | S1 cKO: 161.0 ± 16.1 |  |  |
| **Figure**  **9f** | *Vicia villosa agglutinin*  *(VVA)*  density  (mean ± SEM) | ***Genotype***  (Local) | **Adult (12-week-old)**  M1 ctrl: 147.7 ± 8.3 | two-way ANOVA with Šídák's multiple comparisons test | p= 0.0544  (ns) |
|  |  |  | M1 cKO: 112.1 ± 5.5 |  |  |
|  |  |  | S1 ctrl: 220.4 ± 16.2 | two-way ANOVA with Šídák's multiple comparisons test | p= 0.1044  (ns) |
|  |  |  | S1 cKO: 250.8 ± 6.3 |  |  |
| **Figure**  **9g** | PV+ VVA+  density  (mean ± SEM) | ***Genotype***  (Local) | **Adult (12-week-old)**  M1 ctrl: 48.9 ± 7.2 | two-way ANOVA with Šídák's multiple comparisons test | p=0.4484  (ns) |
|  |  |  | M1 cKO: 36.4 ± 2.7 |  |  |
|  |  |  | S1 ctrl: 71.4 ± 8.7 | two-way ANOVA with Šídák's multiple comparisons test | p= 0.0848  (ns) |
|  |  |  | S1 cKO: 94.9 ± 9.7 |  |  |
| **Figure**  **9g** | PV+ VVA+  percentage  (mean ± SEM) | ***Genotype***  (Local) | **Adult (12-week-old)**  M1 ctrl: : 44.6% ± 1.4 | two-way ANOVA with Šídák's multiple comparisons test | p= 0.0081  (**) |
|  |  |  | M1 cKO: 34.8% ± 2.3 |  |  |
|  |  |  | S1 ctrl: 53.7% ± 1.5 | two-way ANOVA with Šídák's multiple comparisons test | p= 0.1479  (ns) |
|  |  |  | S1 cKO: 9.3% ± 2.8 |  |  |
| **Figure**  **9h** | PV subtypes  density  (mean ± SEM) | ***Genotype***  (Local) | **Adult (12-week-old)**  **M1**  PV+ VVA+: 48.9 ± 7.2 (ctrl), PV+ VVA+: 36.4 ± 2.7 (cKO) | two-way ANOVA with Šídák's multiple comparisons test | p= 0.3793  (ns) |
|  |  |  | PV+ VVA-: 64.7 ± 8.8 (ctrl), PV+ VVA-: 72.6 ± 4.6 (cKO) |  | p= 0.7926  (ns) |
|  |  |  | PV- VVA+: 100.2 ± 7.2 (ctrl),  PV- VVA+: 73.2 ± 6.4 (cKO) |  | p= 0.0263  (*) |
| **Figure**  **9h** | PV subtypes  density  (mean ± SEM) | ***Genotype***  (Local) | **Adult (12-week-old)**  **S1**  PV+ VVA+: 71.4 ± 8.7 (ctrl), PV+ VVA+: 94.9 ± 9.7 (cKO) | two-way ANOVA with Šídák's multiple comparisons test | p=0.2707  (ns) |
|  |  |  | PV+ VVA-: 67.0 ± 7.5 (ctrl), PV+ VVA-: 69.5 ± 10.8 (cKO) |  | p= 0.9978  (ns) |
|  |  |  | PV- VVA+: 151.6 ± 15.8 (ctrl),  PV- VVA+: 143.4 ± 8.1 (cKO) |  | p= 0.9343  (ns) |
| **Figure**  **9j** | PV-VVA  density  (correlation coefficient) | ***Genotype***  (Local) | **Adult (12-week-old)**  r= 0.551 (M1 ctrl) | Pearson correlation | p=0.449  (ns) |
| **Figure**  **9k** |  |  | r= -0.828 (M1 cKO) | Pearson correlation | p=0.172  (ns) |
| **Figure**  **9l** |  |  | r= 0.422 (S1 ctrl) | Pearson correlation | p=0.578  (ns) |
| **Figure**  **9m** |  |  | r= 0.077 (S1 cKO) | Pearson correlation | p=0.923 (ns) |
| **Figure**  **10c** | PV interneuron  distribution  (mean ± SEM) | ***Genotype***  ***Laminar Distribution***  (Local) | **Adult (12-week-old)**  **PV M1**  L1: 0 ± 0 (ctrl),  L1: 0 ± 0 (cKO) | two-way ANOVA with Šídák's multiple comparisons test | p= >0.9999  (ns) |
|  |  |  | L2/3: 143.9 ± 15.8 (ctrl),  L2/3: 125.3 ± 15.2 (cKO) | two-way ANOVA with Šídák's multiple comparisons test | p=0.8536  (ns) |
|  |  |  | L5: 132.7 ± 21.6 (ctrl),  L5: 170.6 ± 13.1 (cKO) | two-way ANOVA with Šídák's multiple comparisons test | p=0.2076  (ns) |
|  |  |  | L6a: 93.2 ± 10.7 (ctrl),  L6a: 91.3 ± 18.8 (cKO) | two-way ANOVA with Šídák's multiple comparisons test | p= >0.9999  (ns) |
|  |  |  | L6b: 26.5 ± 7.7 (ctrl),  L6b: 18.6 ± 6.5 (cKO) | two-way ANOVA with Šídák's multiple comparisons test | p=0.9961  (ns) |
| **Figure**  **10d** | *Vicia villosa agglutinin*  (VVA)  distribution  (mean ± SEM) | ***Genotype***  ***Laminar Distribution***  (Local) | **Adult (12-week-old)**  **VVA M1**  L1: 0 ± 0 (ctrl),  L1: 0 ± 0 (cKO) | two-way ANOVA with Šídák's multiple comparisons test | p= >0.9999  (ns) |
| **Figure**  **10d** | *Vicia villosa agglutinin*  (VVA)  distribution  (mean ± SEM) | ***Genotype***  ***Laminar Distribution***  (Local) | L2/3: 205.1 ± 13.2 (ctrl), L2/3: 154.2 ± 15.3 (cKO) | two-way ANOVA with Šídák's multiple comparisons test | p= 0.0028  (**) |
|  |  |  | L5: 214.6 ± 12.6 (ctrl),  L5: 159.2 ± 12.4 (cKO) | two-way ANOVA with Šídák's multiple comparisons test | p= 0.0011  (**) |
|  |  |  | L6a: 120.5 ± 10.8 (ctrl), L6a: 107.8 ± 5.1 (cKO) | two-way ANOVA with Šídák's multiple comparisons test | p= 0.8803  (ns) |
|  |  |  | L6b: 2.0 ± 2.0 (ctrl),  L6b: 0 ± 0 (cKO) | two-way ANOVA with Šídák's multiple comparisons test | p= >0.9999  (ns) |
| **Figure**  **10e** | *PV subtypes*  *(PV+ VVA+)*  distribution  (mean ± SEM) | ***Genotype***  ***Laminar Distribution***  (Local) | **Adult (12-week-old)**  **PV+ VVA+ M1**  L1: 0 ± 0 (ctrl),  L1: 0 ± 0 (cKO) | two-way ANOVA with Šídák's multiple comparisons test | p= >0.9999  (ns) |
|  |  |  | L2/3: 72.5 ± 9.8 (ctrl),  L2/3: 48.8 ± 6.3 (cKO) | two-way ANOVA with Šídák's multiple comparisons test | p= 0.0562  (ns) |
|  |  |  | L5: 56.6 ± 7.2 (ctrl),  L5: 56.6 ± 10.2 (cKO) | two-way ANOVA with Šídák's multiple comparisons test | p= >0.9999  (ns) |
|  |  |  | L6a: 35.9 ± 5.7 (ctrl),  L6a: 35.9 ± 7.9 (cKO) | two-way ANOVA with Šídák's multiple comparisons test | p= >0.9999  (ns) |
|  |  |  | L6b: 3.8 ± 3.8 (ctrl),  L6b: 0 ± 0 (cKO) | two-way ANOVA with Šídák's multiple comparisons test | p= 0.9962  (ns) |
| **Figure**  **10f** | *PV subtypes*  *(PV+ VVA-)*  distribution  (mean ± SEM) | ***Genotype***  ***Laminar Distribution***  (Local) | **Adult (12-week-old)**  **PV+ VVA- M1**  L1: 0 ± 0 (ctrl),  L1: 0 ± 0 (cKO) | two-way ANOVA with Šídák's multiple comparisons test | p= >0.9999  (ns) |
|  |  |  | L2/3: 77.1 ± 6.1 (ctrl),  L2/3: 81.9 ± 11.2 (cKO) | two-way ANOVA with Šídák's multiple comparisons test | p= 0.9984  (ns) |
|  |  |  | L5: 82.1 ± 18.0 (ctrl),  L5: 131.1 ± 6.6 (cKO) | two-way ANOVA with Šídák's multiple comparisons test | p= 0.0052  (**) |
|  |  |  | L6a: 58.6 ± 9.2 (ctrl),  L6a: 59.4 ±13.4 (cKO) | two-way ANOVA with Šídák's multiple comparisons test | p= >0.9999  (ns) |
| **Figure**  **10f** | *PV subtypes*  *(PV+ VVA-)*  distribution  (mean ± SEM) | ***Genotype***  ***Laminar Distribution***  (Local) | L6b: 25.6 ± 9.9 (ctrl),  L6b: 11.4 ± 5.6 (cKO) | two-way ANOVA with Šídák's multiple comparisons test | p= 0.7992  (ns) |
| **Figure**  **10g** | *PV subtypes*  *(PV- VVA+)*  distribution  (mean ± SEM) | ***Genotype***  ***Laminar Distribution***  (Local) | **Adult (12-week-old)**  **PV- VVA+ M1**  L1: 0 ± 0 (ctrl),  L1: 0 ± 0 (cKO) | two-way ANOVA with Šídák's multiple comparisons test | p= >0.9999  (ns) |
|  |  |  | L2/3: 127.7 ± 11.5 (ctrl), L2/3: 106.1 ± 19.0 (cKO) | two-way ANOVA with Šídák's multiple comparisons test | p= 0.5393  (ns) |
|  |  |  | L5: 157.5 ± 11.5 (ctrl),  L5: 109.7 ± 6.6 (cKO) | two-way ANOVA with Šídák's multiple comparisons test | p= 0.0119  (*) |
|  |  |  | L6a: 81.1 ± 11.7 (ctrl),  L6a: 71.2 ± 15.1 (cKO) | two-way ANOVA with Šídák's multiple comparisons test | p= 0.9670  (ns) |
|  |  |  | L6b: 2.0 ± 2.0 (ctrl),  L6b: 0 ± 0 (cKO) | two-way ANOVA with Šídák's multiple comparisons test | p= >0.9999  (ns) |
| **Figure**  **10j** | *PV interneuron*  distribution  (mean ± SEM) | ***Genotype***  ***Laminar Distribution***  (Local) | **Adult (12-week-old)**  **PV S1**  L1: 0 ± 0 (ctrl),  L1: 0 ± 0 (cKO) | two-way ANOVA with Šídák's multiple comparisons test | p= >0.9999  (ns) |
|  |  |  | L2/3: 110.4 ± 27.9 (ctrl), L2/3: 150.2 ± 16.7(cKO) | two-way ANOVA with Šídák's multiple comparisons test | p= 0.6969  (ns) |
|  |  |  | L4: 234.6 ± 36.2 (ctrl),  L4: 336.8 ± 34.3 (cKO) | two-way ANOVA with Šídák's multiple comparisons test | p= 0.0063  (**) |
|  |  |  | L5: 196.6 ± 16.6 (ctrl),  L5: 259.4 ± 28.4 (cKO) | two-way ANOVA with Šídák's multiple comparisons test | p= 0.2028  (ns) |
|  |  |  | L6a: 97.9 ± 12.1 (ctrl),  L6a: 110.5 ± 12.3 (cKO) | two-way ANOVA with Šídák's multiple comparisons test | p= 0.9987  (ns) |
|  |  |  | L6b: 48.8 ± 7.6 (ctrl),  L6b: 42.4 ± 12.6 (cKO) | two-way ANOVA with Šídák's multiple comparisons test | p= >0.9999  (ns) |
| **Figure**  **10k** | *Vicia villosa agglutinin*  (VVA)  distribution  (mean ± SEM) | ***Genotype***  ***Laminar Distribution***  (Local) | **Adult (12-week-old)**  **VVA S1**  L1: 0 ± 0 (ctrl),  L1: 0 ± 0 (cKO) | two-way ANOVA with Šídák's multiple comparisons test | p= >0.9999  (ns) |
|  |  |  | L2/3: 193.8 ± 14.4 (ctrl), L2/3: 192.1 ± 9.9 (cKO) | two-way ANOVA with Šídák's multiple comparisons test | p= >0.9999  (ns) |
|  |  |  | L4: 605.6 ± 61.9 (ctrl),  L4: 669.1 ± 27.4 (cKO) | two-way ANOVA with Šídák's multiple comparisons test | p= 0.2112  (ns) |
|  |  |  | L5: 288.4 ± 7.9 (ctrl),  L5: 350.7 ± 16.4 (cKO) | two-way ANOVA with Šídák's multiple comparisons test | p= 0.2282  (ns) |
|  |  |  | L6a: 164.7 ± 12.5 (ctrl), L6a: 192.7 ± 5.6 (cKO) | two-way ANOVA with Šídák's multiple comparisons test | p= 0.9252  (ns) |
|  |  |  | L6b: 13.6 ± 2.6 (ctrl),  L6b: 2.4 ± 2.4 (cKO) | two-way ANOVA with Šídák's multiple comparisons test | p= 0.9994  (ns) |
| **Figure**  **10l** | *PV subtypes*  *(PV+ VVA+)*  distribution  (mean ± SEM) | ***Genotype***  ***Laminar Distribution***  (Local) | **Adult (12-week-old)**  **PV+ VVA+ S1**  L1: 0 ± 0 (ctrl),  L1: 0 ± 0 (cKO) | two-way ANOVA with Šídák's multiple comparisons test | p= >0.9999  (ns) |
|  |  |  | L2/3: 53.4 ± 15.4 (ctrl), L2/3: 60.9 ± 8.0 (cKO) | two-way ANOVA with Šídák's multiple comparisons test | p= 0.9999  (ns) |
|  |  |  | L4: 188.5 ± 43.0 (ctrl),  L4: 289.9 ± 38.1 (cKO) | two-way ANOVA with Šídák's multiple comparisons test | p= 0.0023  (**) |
|  |  |  | L5: 100.3 ± 9.8 (ctrl),  L5: 147.5 ± 19.6 (cKO) | two-way ANOVA with Šídák's multiple comparisons test | p= 0.4017  (ns) |
|  |  |  | L6a: 46.9 ± 8.4 (ctrl),  L6a: 60.8 ± 6.1 (cKO) | two-way ANOVA with Šídák's multiple comparisons test | p= 0.9961  (ns) |
|  |  |  | L6b: 5.4 ± 2.2 (ctrl),  L6b: 6.2 ± 6.2 (cKO) | two-way ANOVA with Šídák's multiple comparisons test | p= >0.9999  (ns) |
| **Figure**  **10m** | *PV subtypes*  *(PV+ VVA-)*  distribution  (mean ± SEM) | ***Genotype***  ***Laminar Distribution***  (Local) | **Adult (12-week-old)**  **PV+ VVA- S1**  L1: 0 ± 0 (ctrl),  L1: 0 ± 0 (cKO) | two-way ANOVA with Šídák's multiple comparisons test | p= >0.9999  (ns) |
| **Figure**  **10m** | *PV subtypes*  *(PV+ VVA-)*  distribution  (mean ± SEM) | ***Genotype***  ***Laminar Distribution***  (Local) | L2/3: 74.4 ± 19.5 (ctrl), L2/3: 88.7 ± 10.3 (cKO) | two-way ANOVA with Šídák's multiple comparisons test | p= 0.9740  (ns) |
|  |  |  | L4: 48.1 ± 15.6 (ctrl),  L4: 31.8 ± 8.1 (cKO) | two-way ANOVA with Šídák's multiple comparisons test | p= 0.9510  (ns) |
|  |  |  | L5: 93.2 ± 16.2 (ctrl),  L5: 113.8 ± 26.7 (cKO) | two-way ANOVA with Šídák's multiple comparisons test | p= 0.8651  (ns) |
|  |  |  | L6a: 55.2 ± 4.9 (ctrl),  L6a: 53.6 ± 10.1 (cKO) | two-way ANOVA with Šídák's multiple comparisons test | p= >0.9999  (ns) |
|  |  |  | L6b: 51.9 ± 2.2 (ctrl),  L6b: 39.8 ± 15.9 (cKO) | two-way ANOVA with Šídák's multiple comparisons test | p= 0.9886  (ns) |
| **Figure**  **10n** | *PV subtypes*  *(PV- VVA+)*  distribution  (mean ± SEM) | ***Genotype***  ***Laminar Distribution***  (Local) | **Adult (12-week-old)**  **PV- VVA+ S1**  L1: 0 ± 0 (ctrl),  L1: 0 ± 0 (cKO) | two-way ANOVA with Šídák's multiple comparisons test | p= >0.9999  (ns) |
|  |  |  | L2/3: 138.1 ± 10.2 (ctrl), L2/3: 124.7 ± 13.4 (cKO) | two-way ANOVA with Šídák's multiple comparisons test | p= 0.9958  (ns) |
|  |  |  | L4: 393.4 ± 43.1 (ctrl),  L4: 342.9 ± 27.3 (cKO) | two-way ANOVA with Šídák's multiple comparisons test | p= 0.2724  (ns) |
|  |  |  | L5: 182.2 ± 8.0 (ctrl),  L5: 187.7 ± 24.4 (cKO) | two-way ANOVA with Šídák's multiple comparisons test | p= >0.9999  (ns) |
|  |  |  | L6a: 118.5 ± 14.1 (ctrl), L6a: 129.0 ± 2.3 (cKO) | two-way ANOVA with Šídák's multiple comparisons test | p= 0.9989  (ns) |
|  |  |  | L6b: 9.5 ± 4.0 (ctrl),  L6b: 2.4 ± 2.4 (cKO) | two-way ANOVA with Šídák's multiple comparisons test | p= 0.9999  (ns) |
| **Figure**  **11c** | *PV-VVA*  density  (correlation coefficient) | ***Genotype***  (Local) | **Adult (12-week-old)**  **M1**  L2/3: r= 0.297 (ctrl) | Pearson correlation | p=0.703  (ns) |
|  |  |  | L2/3: r= -0.351 (cKO) | Pearson correlation | p=0.649  (ns) |
|  |  |  | L5: r= 0.211 (ctrl) | Pearson correlation | p=0.789  (ns) |
|  |  |  | L5: r= 0.386 (cKO) | Pearson correlation | p=0.614  (ns) |
|  |  |  | L6a: r= 0.048 (ctrl) | Pearson correlation | p=0.952  (ns) |
|  |  |  | L6a: r= -0.438 (cKO) | Pearson correlation | p=0.562  (ns) |
|  |  |  | L6b: r= -0.549 (ctrl) | Pearson correlation | p=0.451  (ns) |
| **Figure**  **11d** | *PV-VVA*  density  (correlation coefficient) | ***Genotype***  (Local) | **Adult (12-week-old)**  **S1**  L2/3: r= -0.016 (ctrl) | Pearson correlation | p=0.984  (ns) |
|  |  |  | L2/3: r= -0.672 (cKO) | Pearson correlation | p=0.328  (ns) |
|  |  |  | L4: r= 0.038 (ctrl) | Pearson correlation | p=0.962  (ns) |
|  |  |  | L4: r= -0.507 (cKO) | Pearson correlation | p=0.493  (ns) |
|  |  |  | L5: r= -0.925 (ctrl) | Pearson correlation | p=0.075  (ns) |
|  |  |  | L5: r= -0.108 (cKO) | Pearson correlation | p=0.892  (ns) |
|  |  |  | L6a: r= 0.754 (ctrl) | Pearson correlation | p=0.246  (ns) |
|  |  |  | L6a: r= 0.646 (cKO) | Pearson correlation | p=0.354  (ns) |
|  |  |  | L6b: r= -0.533 (ctrl) | Pearson correlation | p=0.467  (ns) |
|  |  |  | L6b: r= -0.475 (cKO) | Pearson correlation | p=0.525  (ns) |
| **Figure**  **12c** | *PV interneuron*  density  (mean ± SEM) | ***Genotype***  (Global) | **Adult (12-week-old)**  CPu ctrl: 64.3 ± 11.9,  CPu cKO: 65.8 ± 5.4 | two-way ANOVA with Šídák's multiple comparisons test | p= >0.9999  (ns) |
|  |  |  | GPe ctrl: 180.8 ± 19.3,  GPe cKO: 194.3 ± 14.9 | two-way ANOVA with Šídák's multiple comparisons test | p= 0.9989  (ns) |
|  |  |  | LP ctrl: 24.0 ± 6.3,  LP cKO: 24.5 ± 3.2 | two-way ANOVA with Šídák's multiple comparisons test | p= >0.9999  (ns) |
|  |  |  | MD ctrl: 15.7 ± 4.0,  MD cKO: 23.0 ± 5.5 | two-way ANOVA with Šídák's multiple comparisons test | p= >0.9999  (ns) |
|  |  |  | TRN ctrl: 765.0 ± 44.7,  TRN cKO: 746.3 ± 47.8 | two-way ANOVA with Šídák's multiple comparisons test | p= 0.9966  (ns) |
|  |  |  | SC ctrl: 341.0 ± 21.3,  SC cKO: 327.3 ± 45.0 | two-way ANOVA with Šídák's multiple comparisons test | p= 0.9989  (ns) |
| **Figure**  **12d** | *PV interneuron*  density  (mean ± SEM) | ***Trajectory***  (Global) | **P21-12 weeks (ctrl)**  P21 LP ctrl: 50.21 ± 14.0  12 weeks LP ctrl: 23.98 ± 6.3 | two-way ANOVA with Šídák's multiple comparisons test | p= 0.9957  (ns) |
|  |  |  | P21 MD ctrl: 62.82 ± 1.6  12 weeks MD ctrl: 15.67 ± 4.0 | two-way ANOVA with Šídák's multiple comparisons test | p=0.8648  (ns) |
|  |  |  | P21 CPu ctrl: 86.31 ± 5.1  12 weeks CPu ctrl: 64.32 ± 11.9 | two-way ANOVA with Šídák's multiple comparisons test | p=0.9980  (ns) |
|  |  |  | P21 GPe ctrl: 397.0 ± 50.8  12 weeks GPe ctrl: 180.8 ± 19.3 | two-way ANOVA with Šídák's multiple comparisons test | p=<0.0001  (****) |
|  |  |  | P21 SC ctrl: 438.7 ± 94.8  12 weeks SC ctrl: 341.0 ± 21.3 | two-way ANOVA with Šídák's multiple comparisons test | p=0.1271  (ns) |
| **Figure**  **12e** | *PV interneuron*  density  (mean ± SEM) | ***Trajectory***  (Global) | **P21-12 weeks (cKO)**  P21 LP cKO: 63.6 ± 10.0  12 weeks LP cKO: 24.5 ± 3.2 | two-way ANOVA with Šídák's multiple comparisons test | p= 0.9796  (ns) |
|  |  |  | P21 MD cKO: 64.3 ± 10.2  12 weeks MD cKO: 23.0 ± 5.5 | two-way ANOVA with Šídák's multiple comparisons test | p= 0.9794  (ns) |
|  |  |  | P21 CPu cKO: 85.3 ± 4.9  12 weeks CPu cKO: 65.8 ± 5.4 | two-way ANOVA with Šídák's multiple comparisons test | p= 0.9997  (ns) |
|  |  |  | P21 GPe cKO: 485.0 ± 117.4  12 weeks GPe cKO: 194.3 ± 14.9 | two-way ANOVA with Šídák's multiple comparisons test | p= <0.0001  (****) |
|  |  |  | P21 SC cKO: 415.7 ± 45.9 12 weeks SC cKO: 327.4 ± 45.0 | two-way ANOVA with Šídák's multiple comparisons test | p=0.4268  (ns) |
| **Figure**  **12f** | *VVA subtypes*  density  (mean ± SEM) | ***Genotype***  (Global) | **Adult (12-week-old)**  CPu VVA+ ctrl: 61.1 ± 3.1 CPu VVA+ cKO: 50.4 ± 7.1 | two-way ANOVA with Šídák's multiple comparisons test | p= 0.2743  (ns) |
|  |  |  | CPu PV- VVA+ ctrl: 25.2 ± 5.6  CPu PV- VVA+ cKO: 21.4 ± 2.9 | two-way ANOVA with Šídák's multiple comparisons test | p=0.8362  (ns) |
| **Figure**  **12g** | *PV subtypes*  percentage | ***Genotype***  (Global) | **Adult (12-week-old)**  CPu PV+ VVA+ ctrl: 55.20% ± 5.5  CPu PV+ VVA+ cKO: 44.86% ± 8.3 | two-way ANOVA with Šídák's multiple comparisons test | p=0.5326  (ns) |
|  |  |  | CPu PV+ VVA- ctrl: 44.80 ± 5.5  CPu PV+ VVA- cKO: 55.13% ± 8.3 | two-way ANOVA with Šídák's multiple comparisons test | p=0.5326  (ns) |
| **Figure**  **12h** | *PV-VVA*  density  (correlation coefficient) | ***Genotype***  (Global) | **Adult (12-week-old)**  CPu ctrl: r= 0.840 | Pearson correlation | p=0.075  (ns) |
|  |  |  | CPu cKO: r= 0.095 | Pearson correlation | p=0.879  (ns) |

**Supplementary table 7.** Summary of data and statistical analyses related to each figure. The table shows the related figure number, type of cell investigated, type of analysis done, related numerical values, and type of statistical tests conducted.
